# Supplementary material for: How IGF-II Binds to the Human Type 1 Insulin-like Growth Factor Receptor
Source: Structure. 2020 Jul 7;28(7):786–798.e6. doi: 10.1016/j.str.2020.05.002 (PMC7343240; doi:10.1016/j.str.2020.05.002)
Supplement: Document S1. Figures S1–S6 and Table S1 [file mmc1.pdf]

Structure, Volume *28*

Supplemental Information

How IGF-II Binds to the Human Type 1 Insulin-like Growth Factor Receptor

Yibin Xu, Nicholas S. Kirk, Hariprasad Venugopal, Mai B. Margetts, Tristan I. Croll, Jarrod J. Sandow, Andrew I. Webb, Carlie A. Delaine, Briony E. Forbes, and Michael C. Lawrence

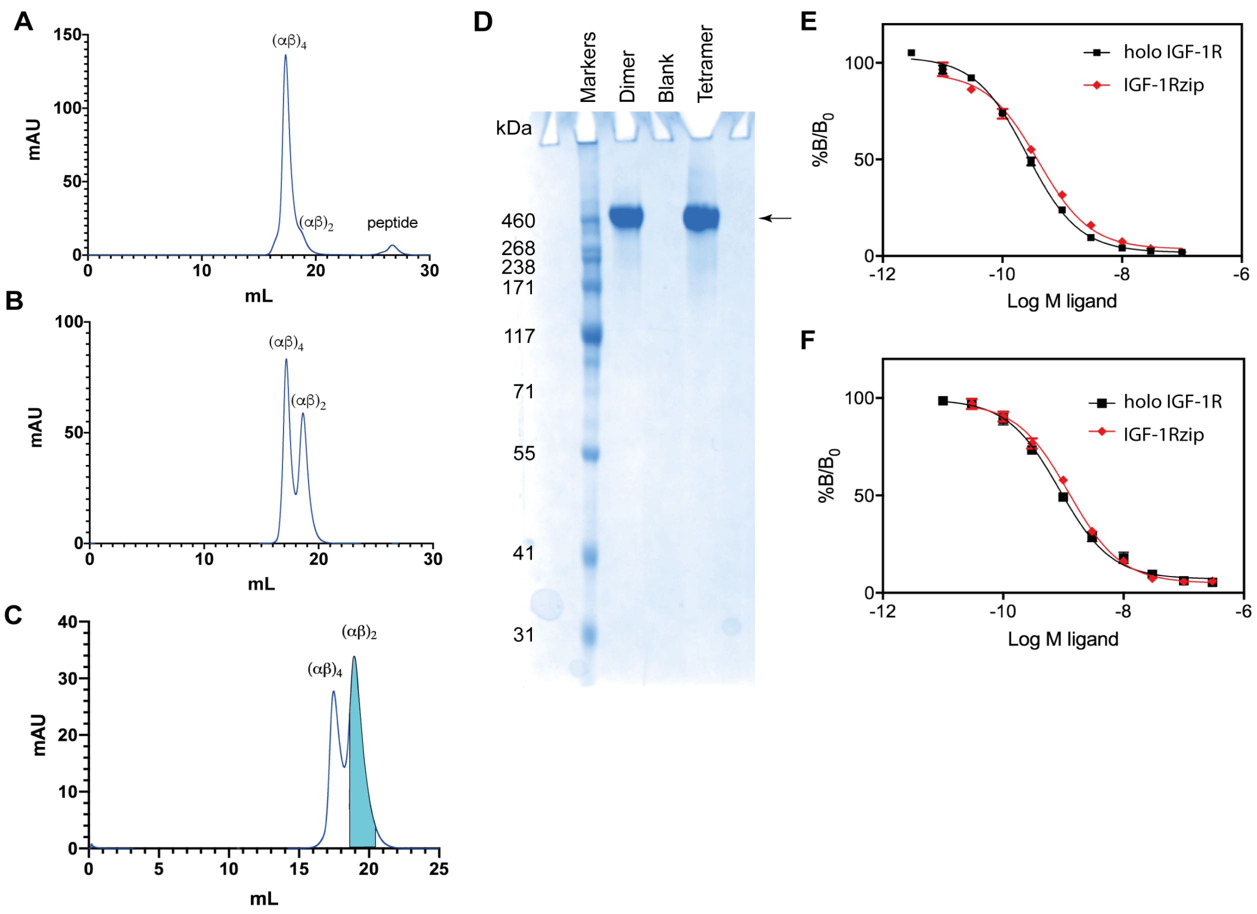

FIGURE S1. Purification and characterization of IGF-1Rzip. Related to STAR methods sections 'Purification of IGF-1Rzip' and 'Receptor competition binding assays'. (**A**) First size-exclusion chromatograph showing a major peak corresponding to the tetrameric form of IGF-1Rzip with a peak shoulder representing the dimeric form of IGF-1Rzip. (**B**) Second size-exclusion chromatograph obtained from the $(\alpha\beta)_2$ shoulder fractions of (A) showing enhanced separation of the dimeric and tetrameric form of IGF-1Rzip. (**C**) Final size-exclusion chromatograph obtained from the $(\alpha\beta)_2$ shoulder fractions of (B) showing further separation of the dimeric and tetrameric form of IGF-1Rzip; fractions highlighted in cyan indicate final pooled fractions of predominantly dimeric protein used for cryoEM analysis in complex with IGF-II. (**D**) Coomassie-stained non-reducing SDS-PAGE gel of the pooled dimeric fractions and pooled tetrameric fractions of IGF-1Rzip from (B) obtained under non-reducing conditions, showing a high level of product purity and indicating that the tetrameric form of the protein product is a non-disulfide-linked dimer of disulfide-linked monomers. The lanes to the left of the "Markers" and right of the "Tetramer" lanes are blank; further lanes cropped beyond these are unrelated to this study. (**E**) Labelled-IGF-I displacement assay comparing IGF-I affinity for IGF-1Rzip (n=9) and for holo IGF-1R (n=9; single individual measurement omitted as aberrant). (**F**) Labelled-IGF-II displacement assay comparing IGF-II affinity for IGF-1Rzip (n=9; four individual measurements omitted as aberrant) and for holo IGF-1R (n=6). For (E) and (F): data are represented as mean ± SEM and, where not visible, error bars are smaller than the marker size.

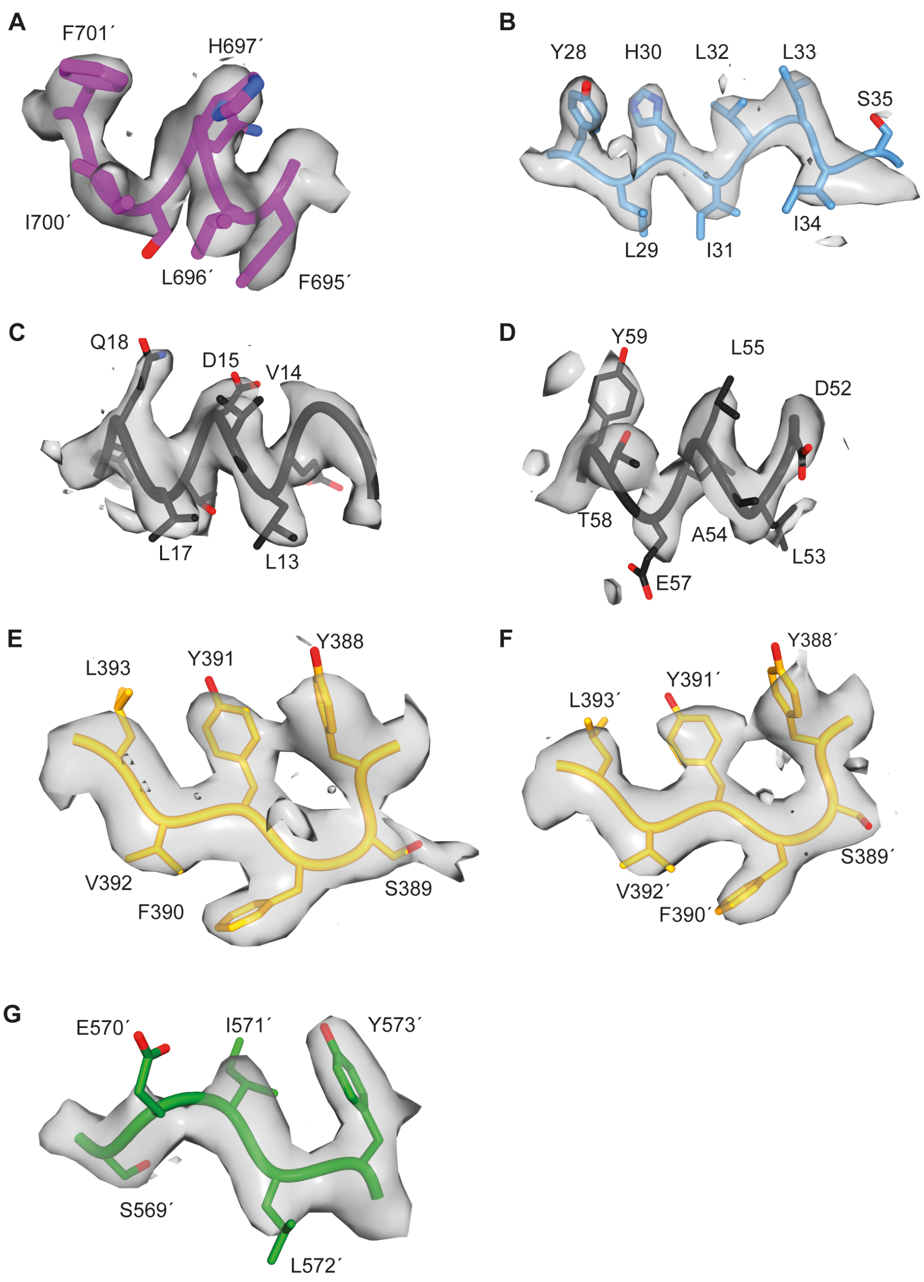

FIGURE S2. Map^{HO} (B_{sharp}= 47.1 Å^2) **and associated atomic model. Related to Figure 4.** (**A**) αCT´ residues Tyr695´ to Val701´. (**B**) Domain L1 residues Tyr28 to Ser35. (**C**) IGF-II B-domain helix residues Val10 to Gly19. (**D**) IGF-I A-domain residues Asp52 to Tyr59. (**E**) Domain L2 residues Ser389 to Leu393. (**F**) Domain L2´ residues Ser389´ to Leu393´. (**G**) Domain F1´ residues Ser569´ to Tyr573´. Surface contours are drawn at a common level of 0.7 units and restricted to lie with 2.5 Å of the associated model segment.´

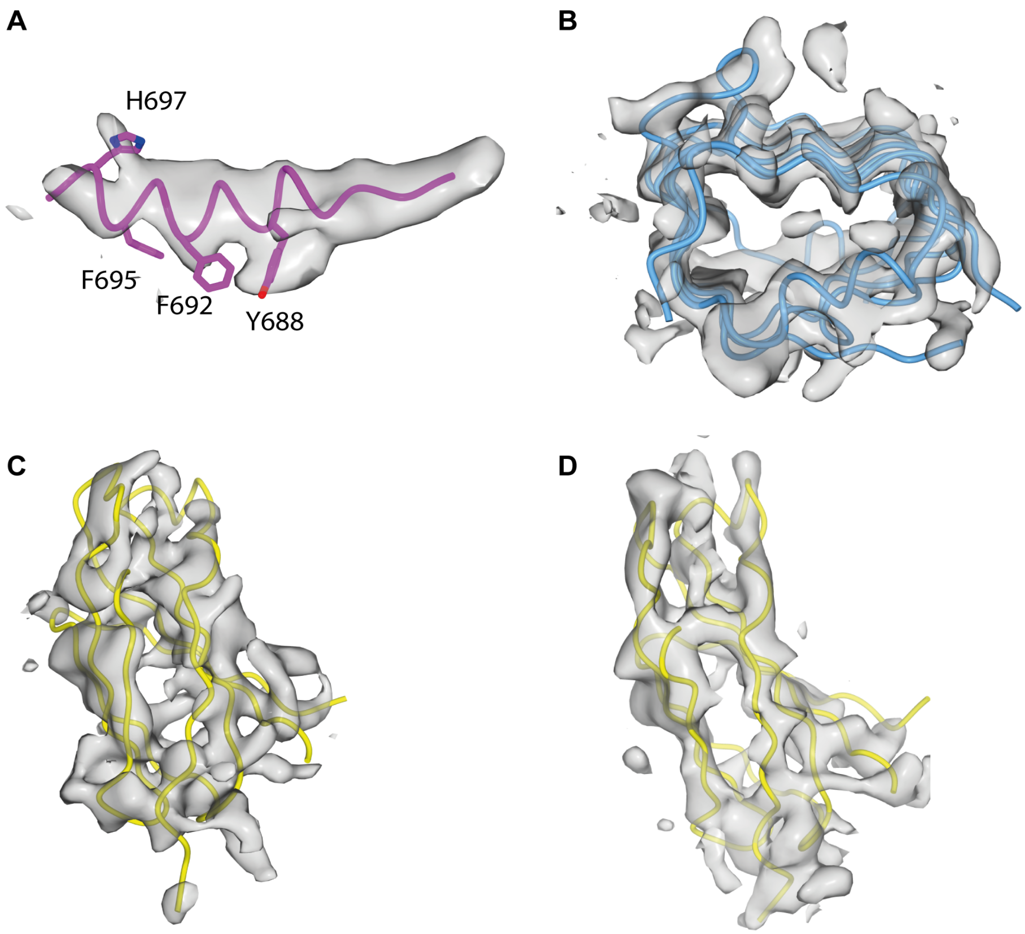

FIGURE S3. Map^{LO} (B_{sharp}= 104.7 Å^2) **and associated atomic model. Related to Figure 4.** (**A**) αCT residues 680-698. (**B**) Domain L1´, residues 1´-150´. (**C**) Domain FnIII-2´, residues 585´-622´ + 720´-800´. (**D**) Domain FnIII-2, residues 585-622 + 720-800. Surface contours are drawn at a common level of 0.5 units and restricted to lie with 3.0 Å of the associated model segment.

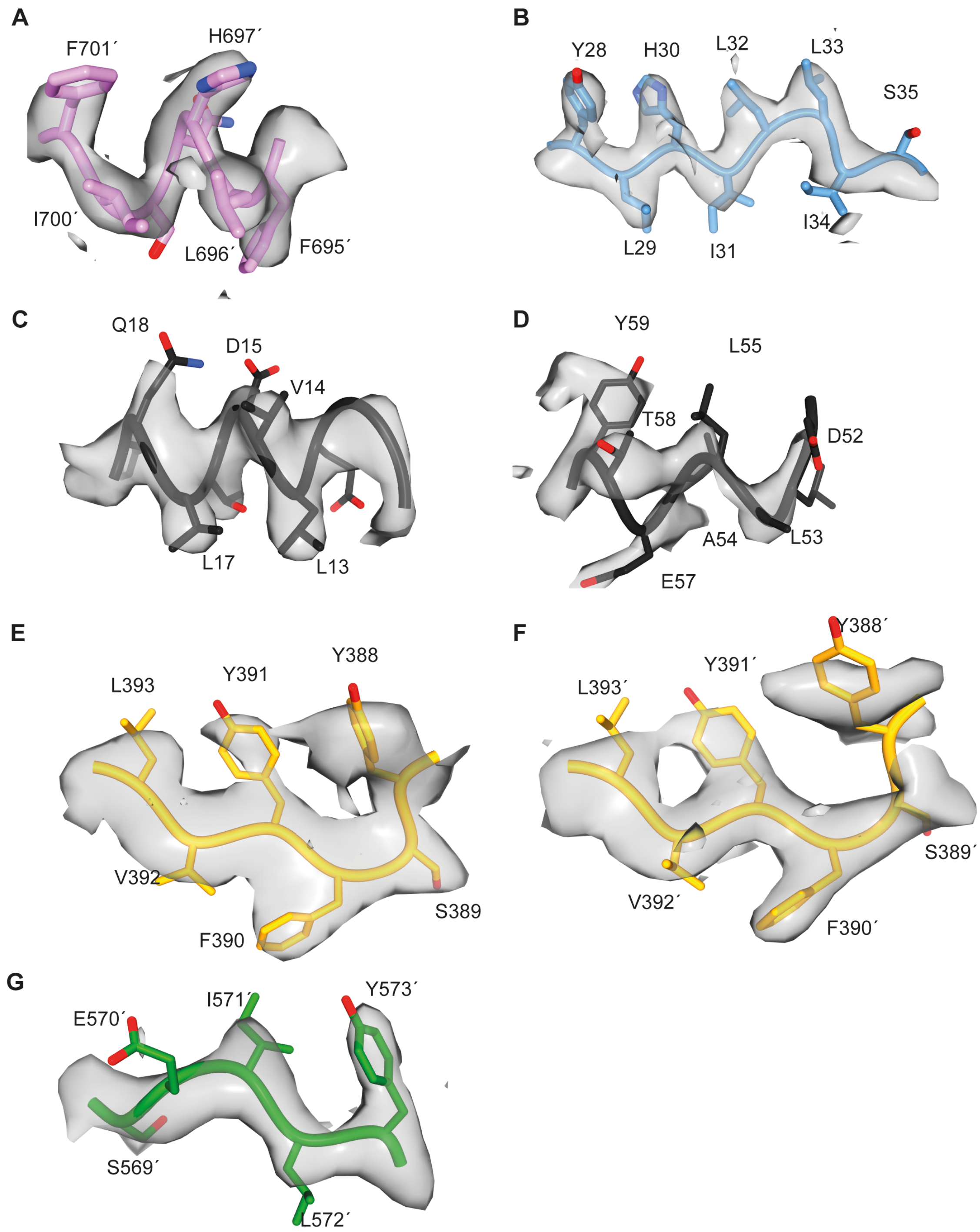

FIGURE S4. Map^{HC} (B_{sharp}= 47.7 Å^2) and associated atomic model. Related to Figure 4. (**A**) αCT´ residues Tyr695´ to Val701´. (**B**) Domain L1 residues Tyr28 to Ser35. (**C**) IGF-II B-domain helix residues Val10 to Gly19. (**D**) IGF-I A-domain residues Asp52 to Tyr59. (**E**) Domain L2 residues Ser389 to Leu393. (**F**) Domain L2´ residues Ser389´ to Leu393´. (**G**) Domain F1´ residues Ser569´ to Tyr573´. Surface contours are drawn at a common level of 0.7 units and restricted to lie with 2.5 Å of the associated model segment.

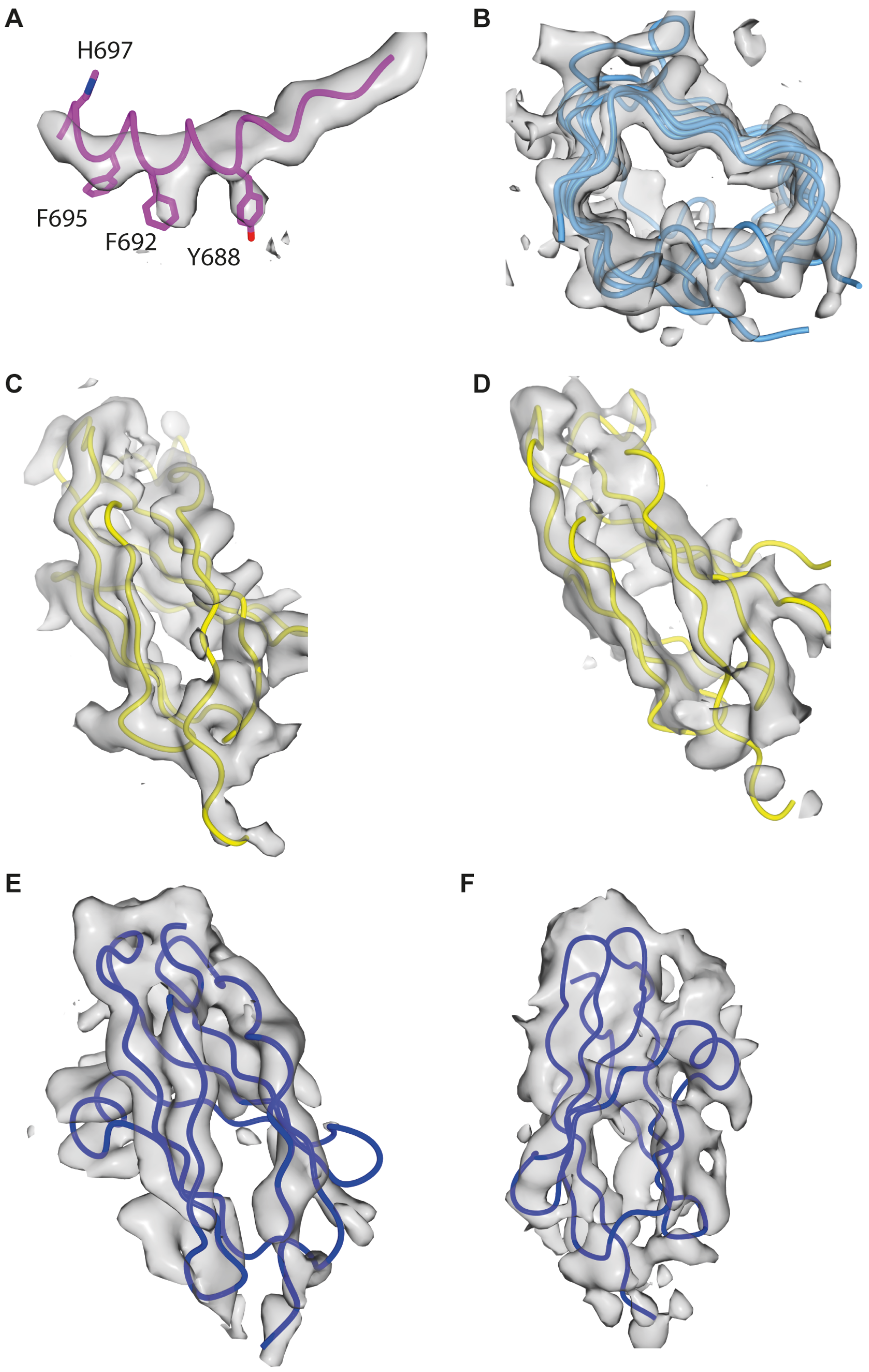

FIGURE S5. Map^{LC} (B_{sharp}= 66.4 Å^2) **and associated atomic model. Related to Figure 4.** (**A**) αCT residues 680-698. (**B**) Domain L1´, residues 1´-150´. (**C**) Domain FnIII-2´, residues 585´-622´ + 720´-800´. (**D**) Domain FnIII-2, residues 585-622 + 720-800. (**E**) Domain FnIII-3´, residues 585´-622´ + 720´-800´. (**F**) Domain FnIII-3, residues 585-622 + 720-800. Surface contours in panels (A-D) are drawn at a common level of 0.5 units and in panels (E,F) at a common level of 0.33 units. All contours restricted to lie with 3.0 Å of the associated model segment.

A

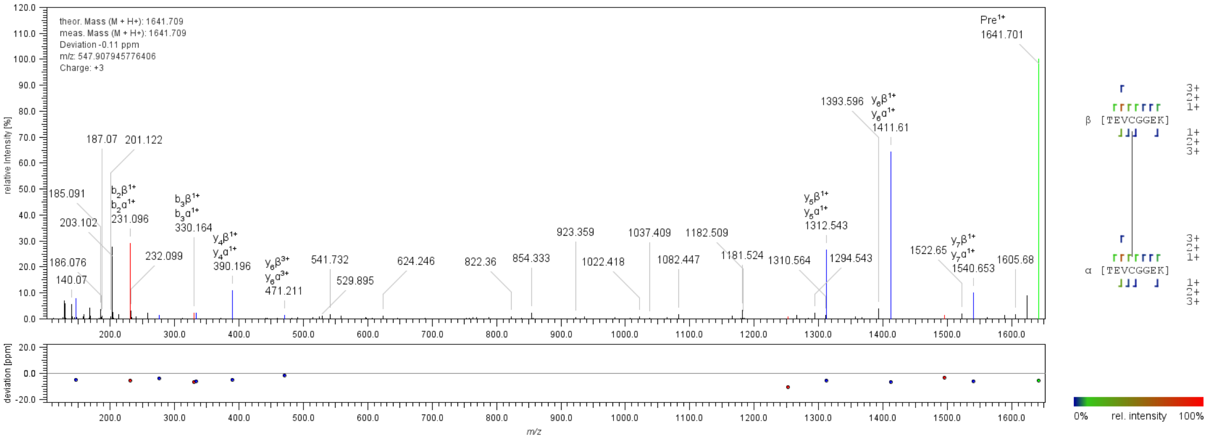

B

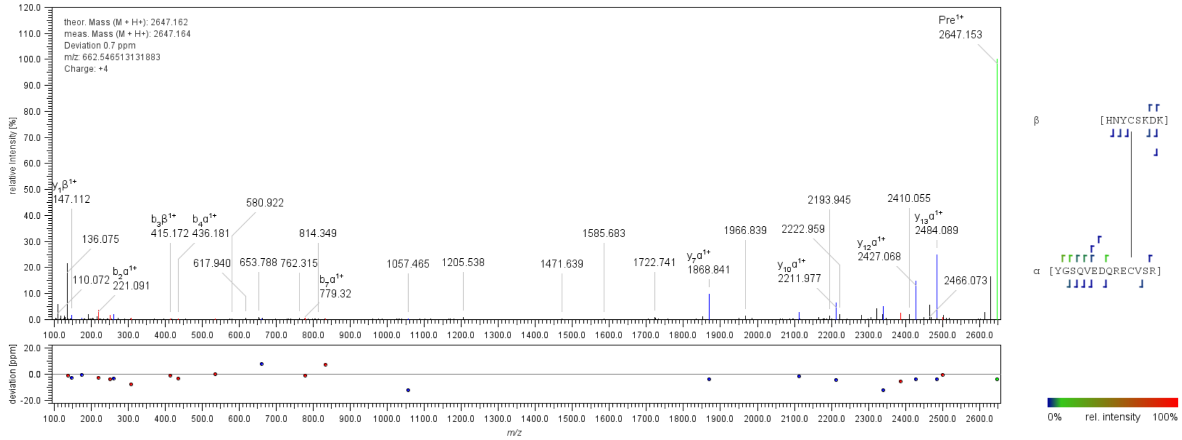

FIGURE S6. Mass spectroscopic identification of peptidic fragments of the IGF-1R ectodomain construct IGF-1RΔβ. Related to STAR Methods section 'Identification of disulfide links using mass spectrometry (MS)'. (**A**) Cys662-Cys662´. (**B**) Cys633-Cys849 (equivalently, Cys633´-Cys849´).

TABLE S1. Residues included in the open- and closed-leg structures of IGF-1Rzip.IGF-II. Related to Figure 4.

PDB	Conformation	Region	Map	Receptor*		IGF-II
				αβ	(αβ)´	
6VWG	Open-leg	Head	Map^{HO}	1-155<br>161-257<br>266-293<br>299-457	299´-511´<br>518´-578´<br>673´-705´	5-32<br>37-62
6VWH	Open-leg	Legs	Map^{LO}	577-633<br>682-704<br>744-799	1´-37´<br>41´-154´<br>162´-300´<br>577´-633´<br>744´-799´	-
6VWI	Closed-leg	Head	Map^{HC}	1-155<br>161-257<br>266-293<br>299-457	299´-511´<br>518´-578´<br>673´-705´	5-32<br>37-62
6VWJ	Closed-leg	Legs	Map^{LC}	580-642<br>682-704<br>744-897	1´-37´<br>41´-154´<br>162´-300´<br>580´-642´<br>744´-897´	-

* The αβ monomer of IGF-1Rzip is defined as that which contributes domain L1 to the IGF-II binding site; the (αβ)´ monomer is that whose domain L1 is ligand-free within this structure.
