# Supplementary material for: How IGF-II Binds to the Human Type 1 Insulin-like Growth Factor Receptor
Source: Structure. 2020 Jul 7;28(7):786–798.e6. doi: 10.1016/j.str.2020.05.002 (PMC7343240; doi:10.1016/j.str.2020.05.002)
Supplement: Document S2. Article plus Supplemental Information [file mmc2.pdf]

Structure

Article

How IGF-II Binds to the Human Type 1 Insulin-like Growth Factor Receptor

Graphical Abstract

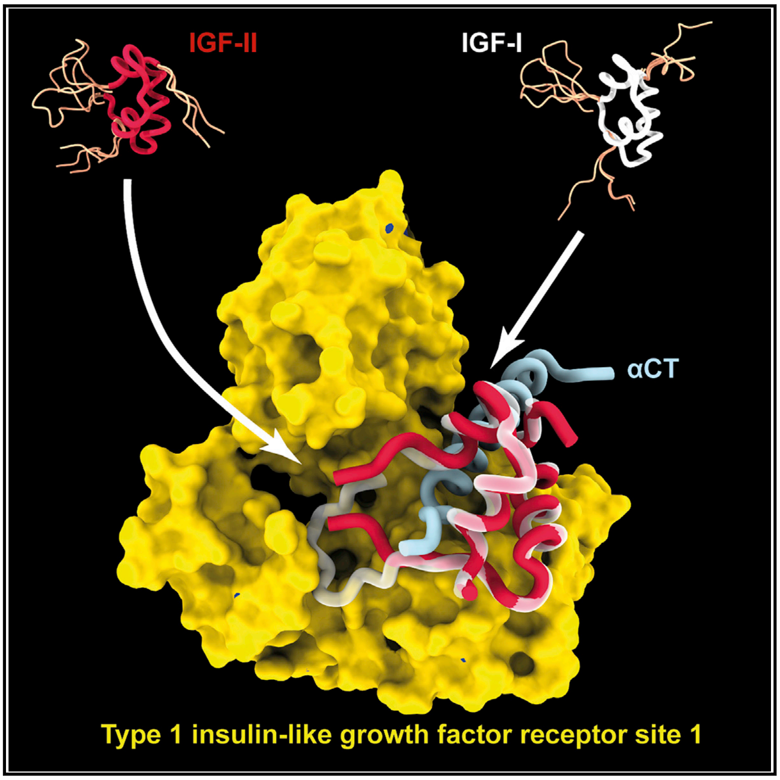

Highlights

- Cryo-EM structure of IGF-II bound to leucine-zippered human IGF-1R ectodomain
- IGF-II C domain interacts with receptor differently to that of IGF-I

Authors

Yibin Xu, Nicholas S. Kirk, Hariprasad Venugopal, ..., Carlie A. Delaine, Briony E. Forbes, Michael C. Lawrence

Correspondence

lawrence@wehi.edu.au

In Brief

Insulin-like growth factors I and II differ in the length of their C domain, the segment that links growth factor B and A domains. Xu et al. show that the different 3D modes of C-domain engagement with type 1 insulin-like growth factor receptor align with their receptor affinity.

Xu et al., 2020, Structure *28*, 786–798
July 7, 2020 © 2020 The Authors. Published by Elsevier Ltd.
https://doi.org/10.1016/j.str.2020.05.002

CellPress

CellPress
OPEN ACCESS

Structure

Article

How IGF-II Binds to the Human Type 1 Insulin-like Growth Factor Receptor

Yibin Xu,[1,2,6] Nicholas S. Kirk,[1,2,6] Hariprasad Venugopal,[3] Mai B. Margetts,[1] Tristan I. Croll,[4] Jarrod J. Sandow,[1,2] Andrew I. Webb,[1,2] Carlie A. Delaine,[5] Briony E. Forbes,[5] and Michael C. Lawrence[1,2,7,*]

[1]Walter and Eliza Hall Institute of Medical Research, Parkville, VIC 3052, Australia
[2]Department of Medical Biology, Faculty of Medicine, Dentistry and Health Sciences, University of Melbourne, Parkville, VIC 3050, Australia
[3]Ramaciotti Centre for Cryo-Electron Microscopy, Monash University, Clayton, VIC 3800, Australia
[4]Cambridge Institute for Medical Research, University of Cambridge, Wellcome Trust/MRC Building, Cambridge CB2 0XY, UK
[5]Flinders Health and Medical Research Institute, College of Medicine and Public Health, Flinders University of South Australia, Bedford Park, SA 5042, Australia
[6]These authors contributed equally
[7]Lead Contact
*Correspondence: lawrence@wehi.edu.au
https://doi.org/10.1016/j.str.2020.05.002

SUMMARY

Human type 1 insulin-like growth factor receptor (IGF-1R) signals chiefly in response to the binding of insulin-like growth factor I. Relatively little is known about the role of insulin-like growth factor II signaling via IGF-1R, despite the affinity of insulin-like growth factor II for IGF-1R being within an order of magnitude of that of insulin-like growth factor I. Here, we describe the cryoelectron microscopy structure of insulin-like growth factor II bound to a leucine-zipper-stabilized IGF-1R ectodomain, determined in two conformations to a maximum average resolution of 3.2 Å. The two conformations differ in the relative separation of their respective points of membrane entry, and comparison with the structure of insulin-like growth factor I bound to IGF-1R reveals long-suspected differences in the way in which the critical C domain of the respective growth factors interact with IGF-1R.

INTRODUCTION

The human type 1 insulin-like growth factor receptor (IGF-1R; Figure 1A) is a disulfide-linked homodimeric member of the receptor tyrosine kinase family (Ullrich et al., 1986; Lemmon and Schlessinger, 2010) that signals into Ras/ERK or PI3K/Akt pathways in response to activation by the insulin-like growth factors I and II (IGF-I and IGF-II) (Adams et al., 2000; Denley et al., 2005; Riedemann and Macaulay, 2006; Laviola et al., 2007; Tao et al., 2007). IGF-1R signaling is involved in normal human growth and development (Denley et al., 2005) and in maintenance of neuroplasticity (Dyer et al., 2016). The bioavailability of IGF-I and IGF-II is controlled by six insulin-like growth factor-binding proteins (Baxter, 2014), and IGF-II is sequestered by the membrane-anchored type 2 insulin-like growth factor receptor (IGF-2R) that can also influence signaling via G-protein interaction (El-Shewy and Luttrell, 2009). The affinity of IGF-II for IGF-1R is reported to be up to an order of magnitude lower than that of IGF-I (Pandini et al., 2002; Surinya et al., 2008; Henderson et al., 2015; Macháčková et al., 2019), with the lower affinity appearing to arise at least in part from differences in the length and amino acid composition of the C domains of the respective growth factors (Denley et al., 2004; Henderson et al., 2015; Hexnerová et al., 2016) (Figure 1B). IGF-1R itself is closely related in structure to its homolog, the human insulin receptor (IR; Figure 1A). The exon-11 minus isoform of IR (IR-A) can signal into growth and/or metabolic pathways in response to IGF-II binding (Belfiore et al., 2017; Holly et al., 2019), with the affinity of IGF-II for IR-A being only slightly weaker than its affinity for IGF-1R (Denley et al., 2005).

Two recent advances have occurred in the structural biology of IGF-1R. The first is the determination of the crystal structure of the IGF-1R ectodomain in apo form (Figure 1C; PDB: 5U8R) at a resolution of 3.0 Å (Xu et al., 2018). The domain arrangement within the apo-IGF-1R ectodomain largely mimics that seen in the structure of the apo-IR ectodomain (McKern et al., 2006; Smith et al., 2010; Croll et al., 2016), but with two salient differences. The first is that the points of membrane entry of the apo-IGF-1R ectodomain are significantly closer together than those of the apo-IR ectodomain (i.e., ~70 Å versus 120 Å). The second is that, in IGF-1R, the critical C-terminal region (αCT) of the receptor α chain interacts not only within domain L1 of the alternate receptor monomer but also with the adjacent domain FnIII-2 of the receptor (Figure 1C). The functional implications of these differences, if any, are unclear. The second advance is the determination of the single-particle cryoelectron microscopy (cryo-EM) structure of the IGF-I bound to murine holoIGF-1R, at an overall resolution of 4.3 Å; this structure is termed herein "holoIGF-1R.IGF-I" (Figure 1D; PDB: 6PYH) (Li et al., 2019). Only a single IGF-I molecule is seen bound to the receptor

 © 2020 The Authors. Published by Elsevier Ltd.
This is an open access article under the CC BY license (http://creativecommons.org/licenses/by/4.0/).

CellPress
OPEN ACCESS

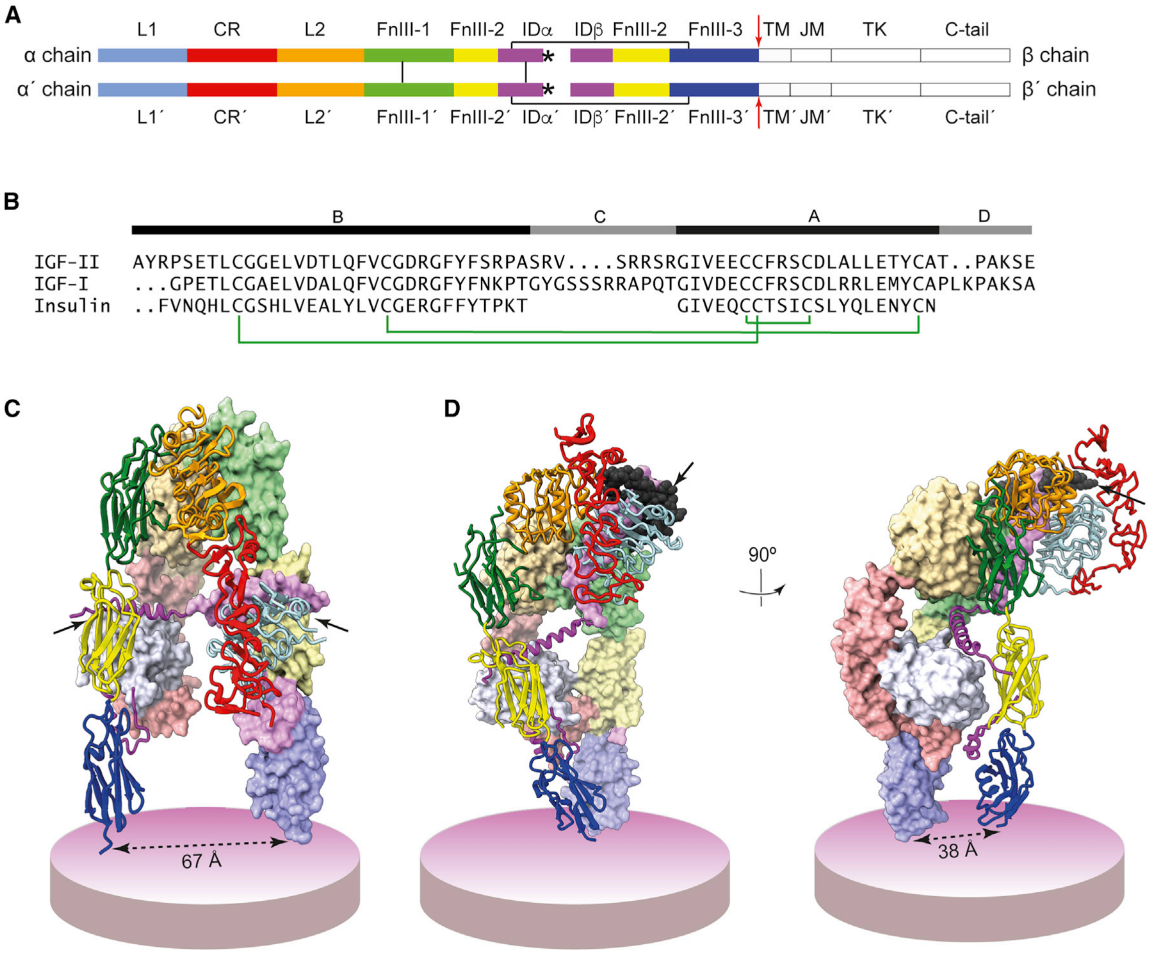

Figure 1. Structure of the IGF-1R Ectodomain in Ligand-Free and IGF-I-Bound Form

(A) Primary structure of IGF-1R (Adams et al., 2000). Each αβ monomer consists of the extracellular domains L1 (first leucine-rich repeat domain), CR (cysteine-rich region), L2 (second leucine-rich repeat domain), FnIII-1, FnIII-2, and FnIII-3 (first, second, and third fibronectin type III domains), followed by the TM (transmembrane domain), JM (juxtamembrane segment), TK (tyrosine kinase domain), and C-tail (C-terminal segment). The insert domain ID (within FnIII-2) contains the αβ cleavage site. The C-terminal segment (αCT) of the α-chain component (IDα) of the ID is indicated by an asterisk; this segment is critical for ligand binding. The α chains are crosslinked by disulfide bonds at least two positions, and each α chain is disulfide linked to its β-chain partner by a single disulfide bond (black lines). Components of the second αβ monomer are indicated by a prime symbol (′). The red arrows indicated the site of leucine-zipper attachment in the ectodomain construct IGF-1Rzip presented here. The domain structure of IR is identical to that of IGF-1R. Panel adapted from Figure 1A of Weis et al. (2018).

(B) Amino acid sequence and domain structure of human IGF-I, IGF-II, and insulin (Adams et al., 2000). Domain boundaries for IGF-I and -II are based here on insulin. Green connectors denote disulfide bonds.

(C) Crystal structure of the apo human IGF-1R ectodomain (PDB: 5U8R [Xu et al., 2018]). Solid arrows indicate the association of the αCT segments with their respective adjacent FnIII-2 domains.

(D) Orthogonal views of the cryo-EM structure of the IGF-I-complexed murine IGF-1R ectodomain (holoreceptor form; PDB: 6PYH [Li et al., 2019]).

In (C) and (D), one receptor monomer is in ribbon depiction, the other in surface depiction (domain coloring is as in A, with lighter shades being used for surfaces), with circular discs depicting the cell membrane. IGF-I in (D) is in atomic-sphere representation (dark gray, arrowed).

ectodomain within holoIGF-1R.IGF-I, consistent with known negative cooperativity of IGF-I binding to IGF-1R (Christoffersen et al., 1994) and with the ligand-to-receptor stoichiometry expected at physiological IGF-I concentrations. IGF-I binding results in extensive conformational change in the membrane-distal region of the IGF-1R ectodomain compared with its apo-ectodomain counterpart (Li et al., 2019), as well as in a bringing-together of the receptor membrane-proximal domains FnIII-3 and FnIII-3′ (Figure 1D). Release of spatial constraints upon these latter domains is understood to be the key event that effects transphosphorylation of the intracellular tyrosine kinase domains (Kavran et al., 2014). The relative domain disposition and mode of ligand binding within holoIGF-1R.IGF-I is similar to that seen in the cryo-EM structure of a single insulin bound to an antibody variable domain (Fv)-complexed, leucine-zippered form of the IR ectodomain (PDB: 6HN4 and 6HN5, termed herein "IRΔβzip.Ins.Fv") (Weis et al., 2018).

Despite these advances, there is currently no three-dimensional (3D) structure of IGF-II bound to IGF-1R or to IR-A. To begin to address this shortcoming, we present here single-particle cryo-EM structures of IGF-II bound to the intact ectodomain of IGF-1R. To obtain these structures, we have used

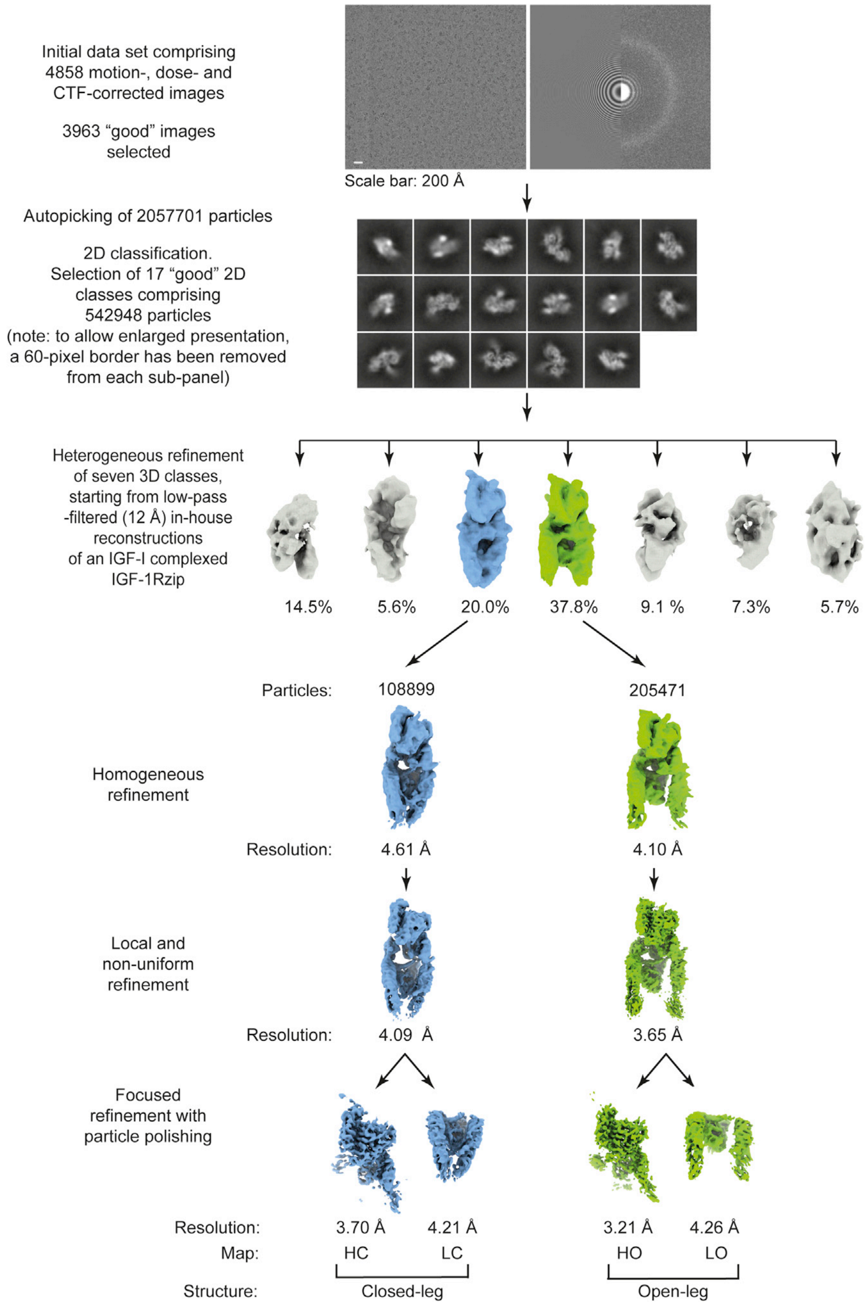

Figure 2. Cryo-EM Data Collection and Reconstruction Scheme

See STAR Methods for full details.

the same leucine-zipper stabilizing technology (Hoyne et al., 2000) that proved successful in the determination of IRΔβzip.Ins.Fv (Weis et al., 2018). This structure (termed herein "IGF-1Rzip.IGF-II," obtained in two distinct 3D conformations) reveals the hitherto unvisualized interaction of the IGF-II C domain with the receptor.

RESULTS

Producing, Purifying, and Characterizing IGF-1Rzip

The construct IGF-1Rzip comprises a 30-residue signal peptide, followed in order by residues 1–905 of the intact holoreceptor, a 33-residue leucine-zipper motif (O'Shea et al., 1991), a 3-residue spacer segment, and an 11-residue c-myc tag. The leucine-zipper motif is thus located at the C-terminal end of the native 11-residue polypeptide spacer that in the intact receptor connects the C-terminal residue Val894 of the final β strand of domain FnIII-3 (Xu et al., 2018) to the N-terminal residue Leu906 of the receptor transmembrane segment (Adams et al., 2000) (Figure 1A). Formation of a coiled-coil leucine-zipper dimer that non-covalently unites the C termini of IGF-1R ectodomain is proposed to act as a "soft restraint" on the spatial separation of the FnIII-3 domains and to provide a mimic of membrane embedding (Hoyne et al., 2000; Weis et al., 2018).

The $(\alpha\beta)_2$ form of IGF-1Rzip was produced by stable expression and secretion from CHO-K1 cells and then purified by a combination of 9E10 antibody-affinity chromatography (Hoogenboom et al., 1991) and three sequential size-exclusion chromatography steps to remove $(\alpha\beta)_4$ forms of the zippered ectodomain wherein the leucine zipper forms between $(\alpha\beta)_2$ dimers rather than within $(\alpha\beta)_2$ dimers (Figures S1A–S1C). The purity of the final protein was high, as assessed by SDS-PAGE analysis (Figure S1D). The affinity of IGF-1Rzip for IGF-I is slightly lower than that of the holoreceptor (IGF-1Rzip: IC_{50} = 0.39 nM, holoIGF-1R: IC_{50} = 0.26 nM, with the 95% confidence intervals on these values being 0.33–0.47 nM and 0.23–0.29 nM, respectively; Figure S1E), and the affinity of IGF-1Rzip for IGF-II is also slightly lower than that of the holoreceptor (IGF-1Rzip: IC_{50} = 1.24 nM, holoIGF-1R: IC_{50} = 0.85 nM, with the 95% confidence intervals on these values being 1.05–1.46 nM and 0.72–1.02 nM, respectively; Figure S1F). Details of the above steps and assays are provided in STAR Methods. We note that the IC_{50} values reported here for IGFs binding to holoIGF-1R differ from those reported by, for example, Macháčková et al. (2019) yet broadly concur with those reported earlier by Surinya et al. (2008); the source of such variation is unclear but may relate to the use of whole-cell versus immunocapture assay formats.

Single-Particle Cryo-EM Reveals Two 3D Classes

The purified IGF-1Rzip homodimer was incubated with an ~1.5-fold stoichiometric ratio of IGF-II to $(\alpha\beta)_2$ dimers in preparation for

CellPress
OPEN ACCESS

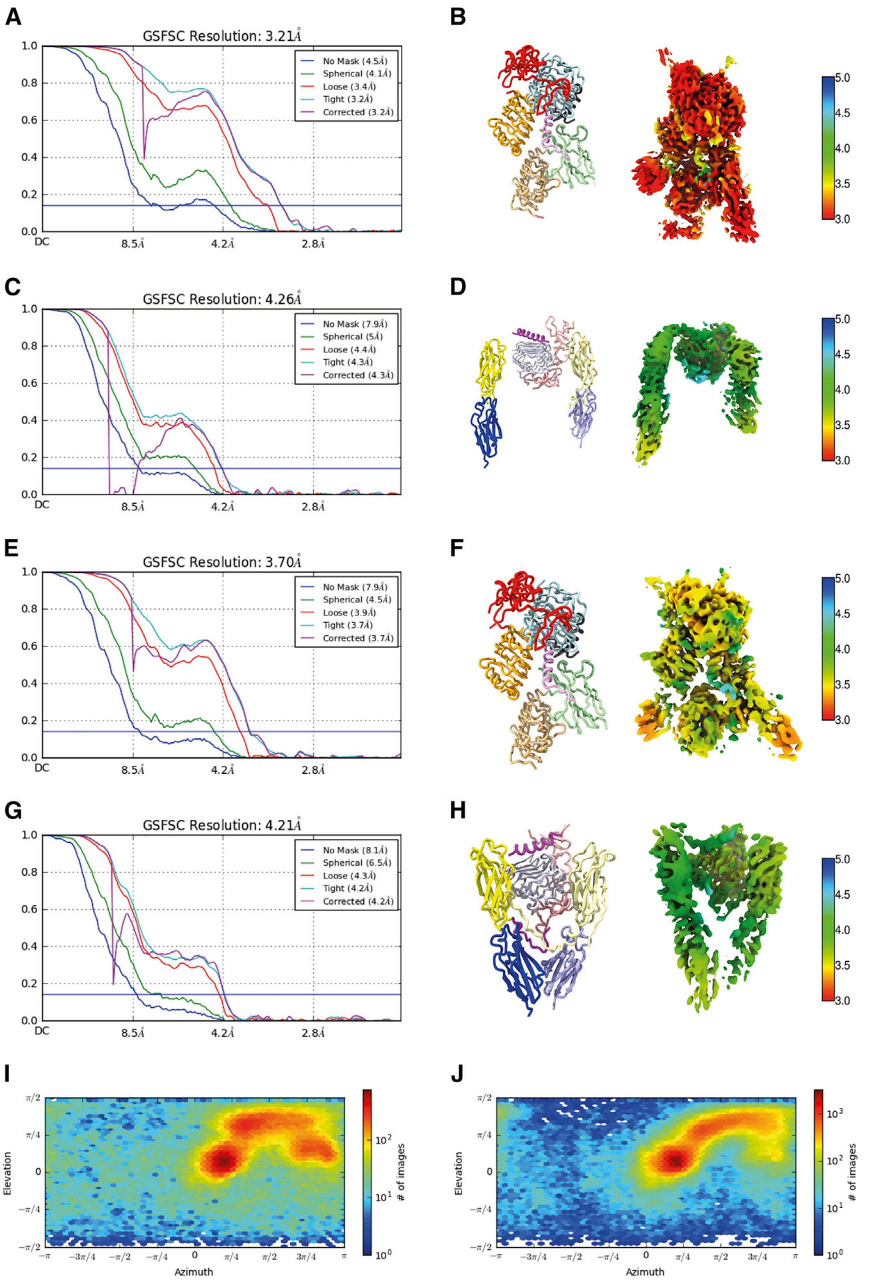

Figure 3. Statistical Analysis of 3D Reconstructions Obtained for the Four Receptor Volumes

(A, C, E, and G) Gold-standard Fourier shell correlation (GSFSC) plots for the half-maps associated the respective reconstruction of Map^{HO} (A), Map^{LO} (C), Map^{HC} (E), and Map^{LC} (G).

(B, D, F, and H) Local resolution of Map^{HO} (B), Map^{LO} (D), Map^{HC} (F), and Map^{LC} (H). Schematics on the left of each panel are included to assist the reader in interpreting the orientation of the local resolution map; colors in these schematics are as in Figure 1C. Domains FnIII-3 and FnIII-3′ in (D) are included for illustrative purposes only and are omitted from the final open-leg model.

(I and J) Angular distribution of the orientation of the particles used in the reconstruction of open (I) and closed (J) conformers of IGF-1Rzip.IGF-II

cryo-EM imaging. 3D classification of cryo-EM-imaged particles yielded two major 3D classes, derived from respectively 37.8% and 20.0% of the total of 542,948 particles subjected to 3D classification (see Figures 2 and 3). The maps corresponding to each of these classes were readily interpretable in terms of the overall configuration of the receptor domains and the location of a single bound ligand. In neither of the maps was any density visible that could be attributed the leucine-zipper element, suggesting either that it is conformationally flexible with respect to the liganded IGF-1R ectodomain or that it adopts a discrete set of conformations that is averaged out during the 3D reconstruction process. The first class (termed the "open-leg structure") displayed a domain configuration similar to that of holoIGF-1R.IGF-I (Li et al., 2019), but with the pair of FnIII-2,3 modules considerably more displaced from each other than in holoIGF-1R.IGF-I. The second class (termed the "closed-leg structure") also displayed a domain configuration similar to that of holoIGF-1R.IGF-I, but with the membrane-proximal ends of the FnIII-3 domains being closer together than they are in holoIGF-1R.IGF-I. Separate focused refinement of the respective membrane-distal ("head") and membrane-proximal ("leg") regions of each the two 3D classes then followed, yielding four map volumes, termed Map^{HO}, Map^{HC}, Map^{LO}, and Map^{LC}, with respective average resolution of 3.21, 3.70, 4.26, and 4.21 Å (see Figures 2 and 3). The map superscript nomenclature employed here denotes the receptor region encompassed by the map (H, head; L, leg) and the receptor conformation associated with that map (O, open leg; C, closed leg). Map^{HO} and Map^{HC} each encompass the bound IGF-II and receptor domains L1, CR, L2, FnIII-1, L2′, FnIII-1′, and αCT′, and Map^{LO} and Map^{LC} each encompass receptor domains FnIII-2, IDα, IDβ, FnIII-3, L1′, CR′, FnIII-2′, IDα′ (excluding αCT′), IDβ′, and FnIII-3′. Domain nomenclature is as in Figure 1A, with the convention that the second monomer—indicated by a prime—is that whose αCT segment (i.e., αCT′) engages IGF-II. Atomic models were then built progressively into each of the four map volumes, using domains from the IGF-1R ectodomain crystal structures (Xu et al., 2018) as starting models and then refining these using real-space refinement protocols. The final structures are depicted in Figure 4A (closed-leg) and Figure 4B (open-leg). Domain FnIII-1 appeared poorly defined in both Map^{HO} and Map^{HC} and domains FnIII-3 and FnIII-3′ appeared poorly defined in Map^{LO}—these domains were thus left unmodeled within the associated structures. Full details of the above processes are presented in STAR Methods, with final statistics in Table 1.

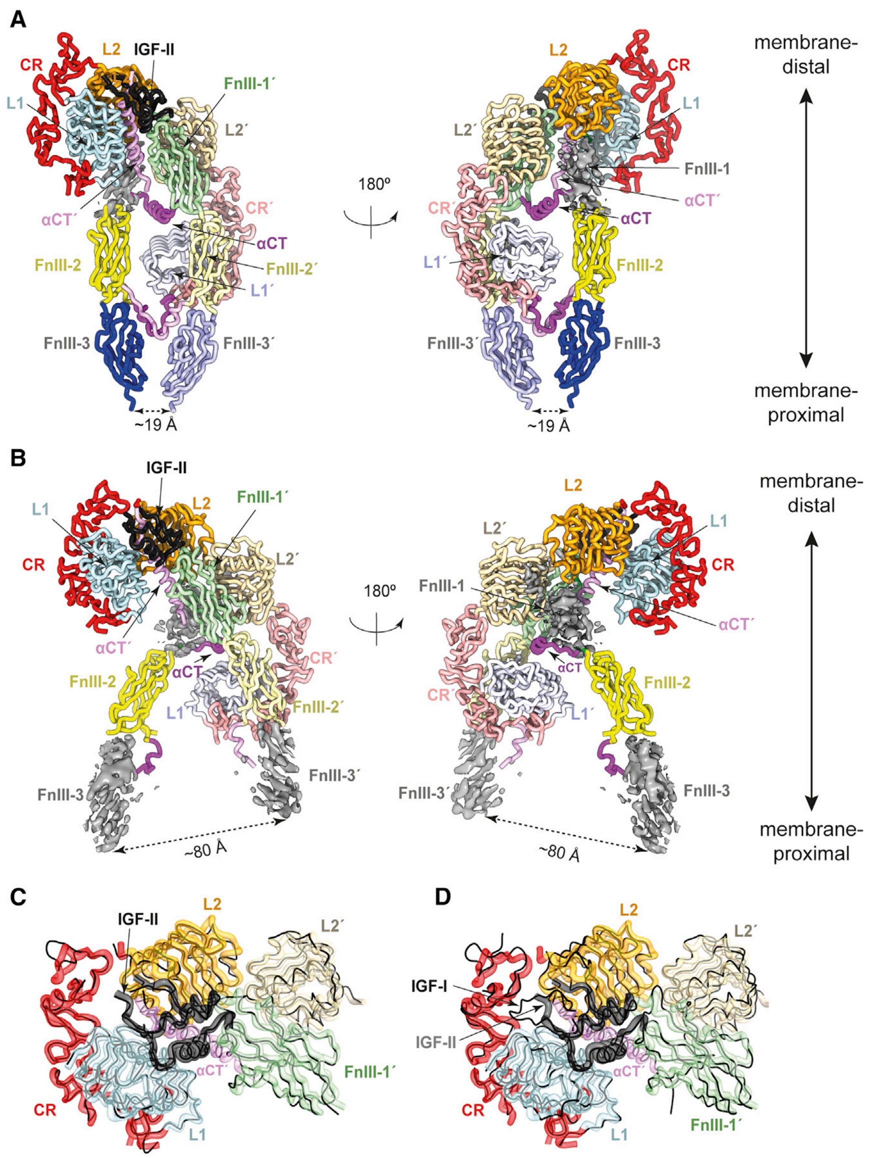

Figure 4. Structure of IGF-1Rzip.IGF-II

(A) Closed-leg conformation of IGF-1Rzip.IGF-II. The single bound IGF-II moiety is in black, with the receptor domains labeled and colored as in Figure 1A; domains of the second (′) chain are in lighter shades. Domain FnIII-1 is excluded from the model due to the poor associated density (gray) within Map^{HC}.

(B) Open-leg conformation of IGF-1Rzip.IGF-II. Domains are labeled and colored as in (A). Domain FnIII-1 and domains FnIII-3 and FnIII-3′ are excluded from the model due to the poor associated density (gray) within Map^{HO} and Map^{LO}, respectively.

(C) Comparison of overall domain disposition within the head regions of the open- and closed-leg conformation of IGF-1Rzip.IGF-II (depicted by fat colored ribbon and thin black ribbon, respectively).

(D) Comparison of overall domain disposition within the head region of the open conformation of IGF-1Rzip.IGF-II and the same region within holoIGF-1R.IGF-I (depicted by fat colored ribbon and thin black ribbon, respectively).

For further details, see also Figures 2, 3, and S2–S5; and Table S1.

Sample potential densities for each constituent domain of the open-leg structure are shown in Figure S2 (head region) and Figure S3 (leg region) and for the closed-leg structure in Figure S4 (head region) and Figure S5 (leg region). A summary of the polypeptide segments included within each structure is provided in Table S1; excluded segments are either poorly defined or absent within the respective associated maps.

We now present descriptions of the atomic models.

IGF-II Interacts with the Receptor Differently to IGF-I

The head regions of open- and closed-leg structures are effectively identical in structure (Figure 4C); hence, the description of the head region that follows will be limited to that seen in the open-leg structure, as its associated map (Map^{HO}) is of higher resolution than that of the closed-leg structure (Map^{HC}). We note, in particular, that domain FnIII-1 (in contrast to domain FnIII-1′) is poorly ordered in both maps and is excluded from both the respective models. The salient features of the head region of IGF-II.IGF-1Rzip are as follows.

(1) A single IGF-II molecule is seen bound within the head region (Figures 4A and 4B), interacting with receptor domains L1, L2, αCT′, and FnIII-1′. IGF-II binding is seen to result in the receptor's L1-CR + (αCT′) module folding away from its location in the apo receptor (i.e., adjacent to domain FnIII-2′) to position the bound IGF-II close to the apex of the receptor and to permit interaction of the growth factor with the membrane-distal part of domain FnIII-1′ (Figures 4A and 4B). This repositioning of the L1-CR + (αCT′) module involves a concomitant outward rotation of domain L2 from its location within the two-fold symmetric $(L2\text{-}[FnIII\text{-}1])_2$ assembly found within the apo ectodomain (Figure 1C). IGF-II binding results further in a repositioning and reconfiguring of the αCT′ segment on

Table 1. Statistics for the Open- and Closed-Leg Structures of IGF-1Rzip.IGF-II[a]

	Open-Leg Structure		Closed-Leg Structure	
	Head	Legs	Head	Legs
Model				
PDB ID	6VWG	6VWH	6VWI	6VWJ
Composition (#):				
Chains	5	2	5	2
Atoms (including hydrogens)	12,813	8,465	12,814	11,807
Protein residues	800	539	800	747
Glycan residues	4	0	4	0
Bonds (RMSD):				
Length (Å) (# > 4σ)	0.005 (0)	0.004(0)	0.005(0)	0.004 (0)
Angles (°) (# > 4σ)	0.8 (0)	0.9 (3)	0.7 (0)	0.8 (0)
MolProbity score	1.77	1.90	1.84	1.77
Clashscore	2.81	7.91	2.42	6.36
Ramachandran plot (%):				
Outliers/allowed/favored	0.00/11.38/88.62	0.76/6.69/92.54	0.00/11.89/88.11	0.41/4.79/94.80
Rotamer outliers (%)	1.40	0.82	1.97	1.20
C^{β} outliers (%)	0.00	0.00	0.00	0.00
Peptide plane (%):				
cis proline/general	3.8/0.0	2.9/0.0	3.8/0.0	1.8/0.0
Twisted proline/general	0.0/0.0	0.0/0.0	0.0/0.0	0.0/0.0
C^{α}BLAM outliers (%)	4.58	3.94	4.58	3.08
ADP:				
Iso/aniso (# atoms)	6,470/0	4,322/0	6,470/0	6,010/0
Protein (min/max/mean)	58/115/80	74/287/125	57/127/91	62/402/140
Glycan (min/max/mean)	75/87/80	–	68/99/85	–
Occupancy (# atoms)				
Occ = 1/0.5/0.0	12,813/0/0	8,465/0/0	12,814/0/0	11,807/0/0
Map				
Resolution (Å): FSC independent half-maps	3.21	4.26	3.70	4.21
Local resolution range (Å)				
Sharpening *B* -factor (Å^2)	47.1	104.7	66.4	47.7
Model versus map				
CC_{mask}	0.72	0.62	0.74	0.65
CC_{box}	0.71	0.70	0.78	0.79
CC_{volume}	0.72	0.61	0.73	0.66
CC individual chains:				
IGF-1Rzip chains A, B	0.70, 0.70	0.63, 0.63	0.73, 0.72	0.66, 0.71
IGF-II	0.68	–	0.72	–
Glycan chains A, B	0.62, 0.63	–	0.64, 0.62	–
Resolution (Å): FSC, masked map versus model @ 0.143	3.12	4.29	3.47	4.02

[a]See Table S1 for further details.

the surface of domain L1, with the αCT′ segment threading through the polypeptide loop formed by the IGF-II C domain (residues 33–40; Figure 1B) and the growth factor helical core. Opening of the IGF-II C-domain loop is facilitated in turn by a folding out of the IGF-II B-domain C-terminal segment away from the B-domain helix. These structural rearrangements within the head region of the receptor and within the ligand reflect those seen within holoIGF-1R.IGF-I (Figure 4D) upon its comparison with the apo-IGF-1R ectodomain structure (Li et al., 2019).

(2) Differences nevertheless emerge in the way in which the IGF-II C domain interacts with the receptor compared with its IGF-I counterpart in the holoIGF-1R.IGF-I structure. In particular, the segment of the C domain of IGF-II

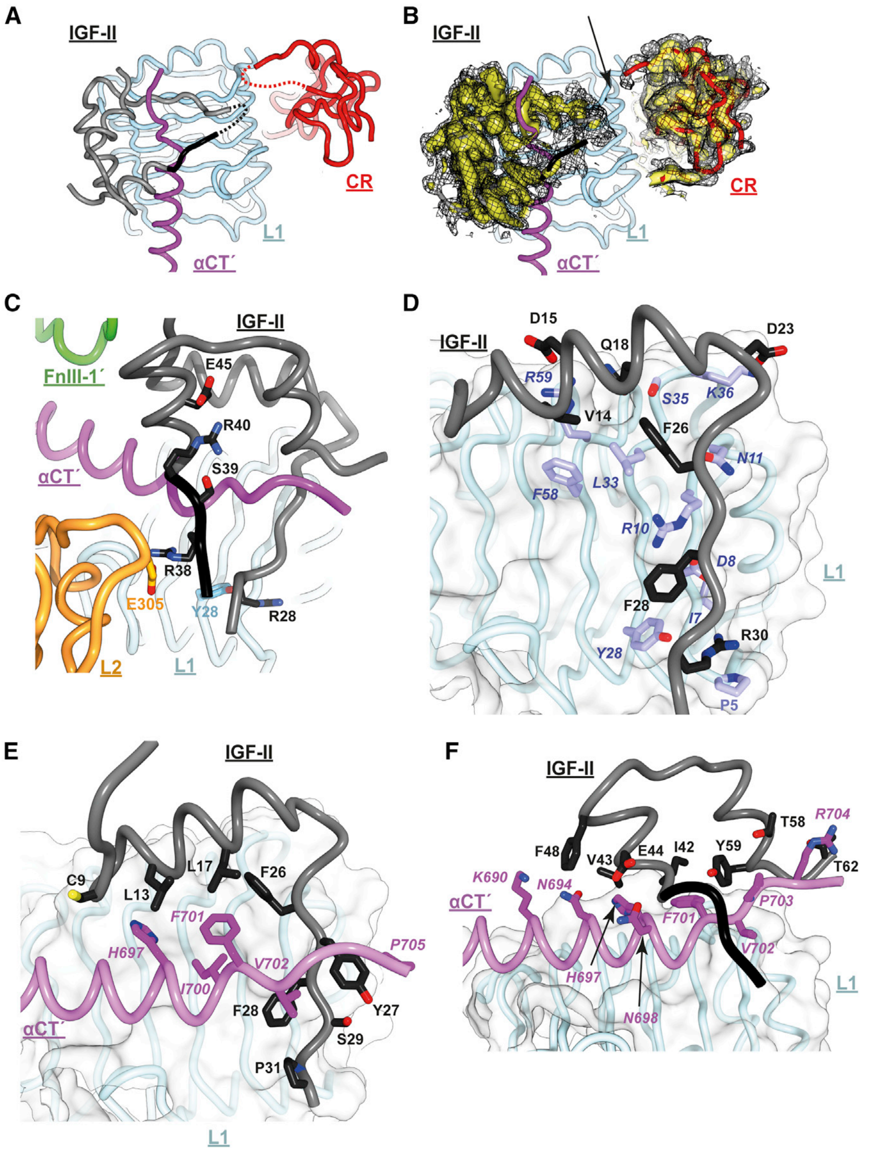

Figure 5. Interaction of IGF-II with the Primary Binding Site of IGF-1R

(A) Disposition of IGF-II on the surface of domain L1. IGF-II B and A domains are in gray ribbon and C domain is in black ribbon; IGF-1R domains are as labeled. Residues 33–36 of IGF-II and residues 258–265 of domain CR are left unmodeled due to weak associated density; their tentative location is indicated by curved dashed lines.

(B) Density associated with (A), contoured at two levels (yellow surface and black mesh representation, respectively) and excluding domain L1 and the αCT′ segment. The absence of density at the IGF-II to CR interface is arrowed.

(C) Detail of the interaction of the IGF-II C domain with IGF-1R and with the IGF-II A domain.

(D) Interaction of the B domain of IGF-II with receptor domain L1.

(E) Interaction of the B domain of IGF-II with IGF-1R αCT′ segment. In (D) and (E), surrounding receptor domains and the C and A domains of IGF-I are omitted for clarity.

(F) Interaction of the A domain of IGF-II with the IGF-1R αCT′ segment.

that is most distal to growth factor core (namely, IGF-II residues 33–36) is disordered, as are the adjacent receptor CR domain residues 258–265 (Figures 4D, 5A, and 5B). By contrast, the C domain of IGF-I in holoIGF-1R.IGF-I is relatively well ordered (Figure 4D), with its distal loop engaging receptor residues Pro5 and Pro256 via IGF-I residue Tyr31 (Li et al., 2019). The IGF-II C domain is four residues shorter than that of IGF-I (Figure 1B) and lacks an aromatic counterpart to IGF-I Tyr31; the IGF-II C domain thus appears too short to form intimate contact with the receptor CR domain. However, the C domain of IGF-II has an arginine at position 40 (threonine in IGF-I; Figure 1B) that is stabilized both by (i) a salt bridge with IGF-II residue Glu45 that lies near the N terminus of the first helix of the IGF-II A domain, and by (ii) a polar interaction with the side chain of the adjacent IGF-II residue Ser39 (Figure 5C). In the solution structure of IGF-II (PDB: 1IGL [Torres et al., 1995]), the C domain of the growth factor is highly mobile and appears to lack these intramolecular interactions.

(3) The folded-out B chain of IGF-II also appears to be stabilized by an interaction between residue IGF-II residue Arg30 (lysine in IGF-I, the side chain of which is unresolved in holoIGF-1R.IGF-I) and the hydroxyl side-chain group of IGF-1R residue Tyr28 (Figure 5D). An additional C-domain stabilizing interaction is a possible salt bridge between IGF-II residue Arg38 and IGF-1R residue Glu305 (modeled but relatively unclear in Map^{HO}) (Figure 5C). Arg38 is replaced by proline in IGF-I (assuming the equivalent residue to be that three residues upstream of the common Gly-Ile-Val motif shared by IGF-II and IGF-I) and is unmodeled in holoIGF-1R.IGF-I, both suggesting the absence of an equivalent receptor interaction for IGF-I.

CellPress
OPEN ACCESS

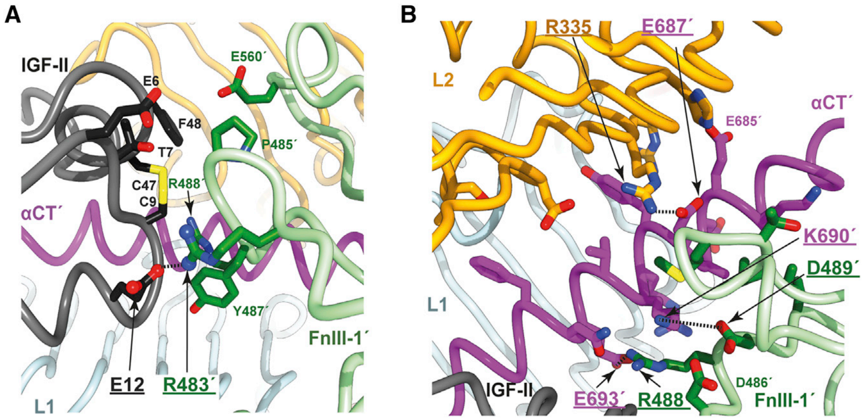

Figure 6. Interaction of IGF-II with Domain FnIII-1′ and Associated Interactions within IGF-1R

(A) Detail of the interaction of IGF-II and domain FnIII-1′, highlighting, in particular, the likely salt bridge (dashed line) between IGF-II residue Glu12 and IGF-1R residue R483′ (boxed labels with ball-and-stick side-chain atoms).

(B) Detail of the interaction of the reconfigured αCT′ helix with surrounding IGF-1R domains L2 and FnIII-1′, highlighting, in particular, salt-bridge formation between residues R355 and Glu687′, between residues Asp489′ and Lys690′, and between residues Glu693′ and Arg488′.

(4) The interaction of the IGF-II B domain with the receptor is extensive and mediated by the side chains of IGF-II residues Cys9, Leu13, Val14, Asp15, Leu17, Gln18, Asp23, Phe26, Tyr27, Phe28, Ser29, and Arg30, the side chains of receptor domain L1 residues Pro5, Ile7, Asp8, Arg10, Asn11, Leu33, Ser35, Ly36, Phe58, and Arg59, and the side chains of receptor αCT′ residues His697′, F701′, Val 702′, and Pro705′ (Figures 5D and 5E). The IGF-II A domain contacts the receptor αCT′ segment (but not domain L1), with the interaction mediated by the side chains of IGF-II residues Ile42, Val43, Glu44, Phe48, Thr58, Tyr59, and Thr62 and the side chains of receptor αCT′ residues Lys690′, Glu694′, His697′, Asn698′, Phe701′, Val702′, Pro703′, and Arg704′ (Figure 5F). Of the IGF-II B-domain residues listed above, all except Ser29 and Arg30 are conserved in IGF-I, whereas of the IGF-II A-domain residues listed above, only Ile42, Val43, Phe48, and Tyr59 are conserved in IGF-I.

(5) The interaction of IGF-II with domain FnIII-1′ involves, inter alia, the formation of a salt bridge between IGF-II residue Glu12 (lying close to the N terminus of the ligand's B-domain helix) and IGF-1R residue Arg483′ (lying within the canonical BC loop of domain FnIII-1′) (Figure 6A). Glu12 is highly conserved within both IGF-II and IGF-I sequences, as is Arg483′ within IGF-1R sequences. The presence of this salt bridge aligns with the observation that mutation of the IGF-II residue Glu12 to alanine results in a ~2-fold reduction in IC_{50} for IGF-II binding to solubilized IGF-1R ectodomain and a ~6-fold reduction in IC_{50} for IGF-II binding to surface-expressed holoIGF-1R (Alvino et al., 2009). An equivalent interaction is seen in the deposited coordinates of holoIGF-1R.IGF-I (between IGF-I Glu9 and IGF-1R Arg483, human sequence numbering), but is not remarked upon in the associated paper (Li et al., 2019).

(6) The reconfigured αCT′ segment makes extensive interactions with domains L2′ and FnIII-1′. As far as can be discerned, these interactions mimic those seen in holoIGF-1R.IGF-I but can now be described with more confidence, given the higher resolution of our structure (3.21 Å) compared with that of holoIGF-1R.IGF-I (4.3 Å) (Li et al., 2019). In particular, salt bridges are here seen to be possible between residues Glu687′ (αCT′) and Arg335 (domain L2), between residues Glu693′ (αCT′) and Arg488′ (domain FnIII-1′), and between residues Lys690′ (αCT′) and Asp489′ (domain FnIII-1′) (Figure 6B).

Two Distinct Conformations for the "Legs"

The leg regions of both the closed- and open-leg conformations are less well resolved than their corresponding head regions. Within the closed-leg structure, domains L1′, FnIII-2, FnIII-2′, FnIII-3, and FnIII-3′ can all be tentatively modeled, as can the αCT segment. The respective C termini of domains FnIII-3 and FnIII-3′ are closer together in the closed-leg structure (separation of ~12 Å) than in holoIGF-1R.IGF-I (separation of ~38 Å; Figure 1D), but, as in holoIGF-1R.IGF-I, there is no direct interaction between the domains FnIII-3 and FnIII-3′ (Figure 7A). The L1′-CR′ module of the closed-leg structure appears to contact both domains FnIII-2 and FnIII-2′, in a fashion similar to that seen in the cryo-EM structure of holoIGF-1R.IGF-I (Figure 7A). The αCT segment also adopts a conformation on the surface of domain L1 similar to that observed both in the apo-ectodomain crystal structure (Xu et al., 2018) and in holoIGF-1R.IGF-I (Li et al., 2019). However, our modeled conformation for the IDα segments immediately C-terminal to the respective α-β and α′-β′ disulfide bonds is different to that modeled in holoIGF-1R.IGF-I. In our structure, these parts of the IDα segments "cross over" from their parent FnIII-2 domain to the alternate FnIII-2 domain (Figure 7A)—in holoIGF-1R.IGF-I, the equivalent part of the single modeled IDα segment undergoes a reverse turn to reassociate with its parent FnIII-2 (Figure 7A). We suggest that our pathway for this segment is more "natural," in that it appears to direct the C termini of these IDα segments toward the N termini of their respective downstream IDα αCT segments.

The quality of the map corresponding to the open-leg structure is lower than that of the closed-leg structure, permitting only rigid-body positioning of domains into the map followed by limited real-space refinement (Figure 7B). Secondary-structure elements within the density for the domains FnIII-3 and FnIII-3′ were not readily discerned, leading to preclusion of these domains from the open-leg atomic model. Surprisingly, neither of the two FnIII-2 leg modules appear to make contact with the ligand-free (L1′-CR′) + αCT module in the open-leg structure

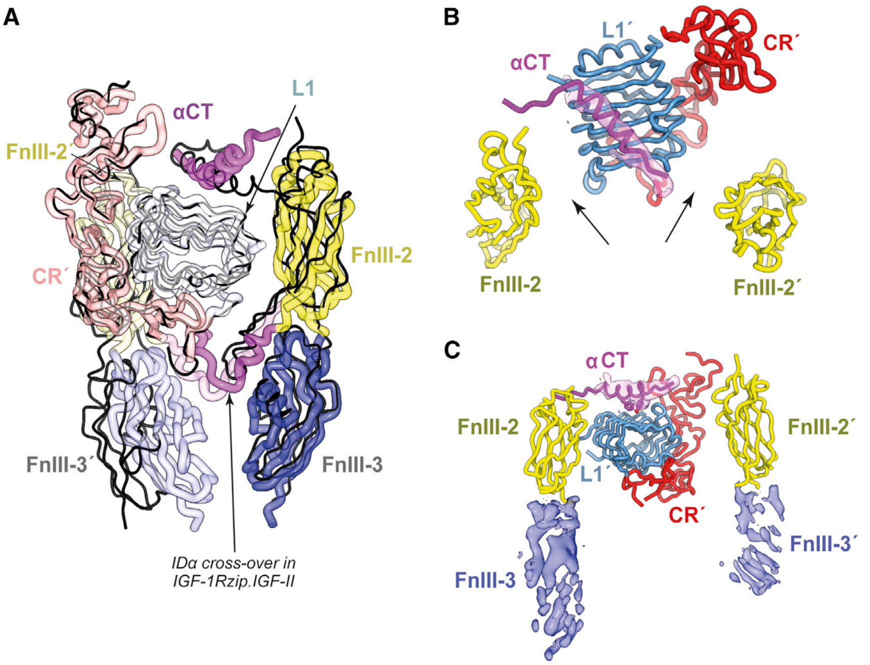

Figure 7. Conformation of the Leg Region of IGF-1Rzip.IGF-II

(A) The closed-leg conformation of IGF-1Rzip.IGF-II (thick ribbons, colored as in Figure 1A), overlaid with the structure of the equivalent region of the structure holoIGF-1R.IGF-I (solid black line, PDB: 6PYH [Li et al., 2019]). The difference in the relative dispositions of the FnIII-3 domains within each structure is apparent. Also indicated is the manner in which the N-terminal regions of each IDa segment in IGF-1Rzip.IGF-II "crosses over" from one FnIII-2 domain to the other; in holoIGF-1R.IGF-1R, the single modeled segment is directed spatially back to its parent FnIII-2 domain.

(B) The leg domains of the open-leg conformation of IGF-1Rzip.IGF-II, viewed in a direction approximately parallel to the pseudo two-fold axis relating domains FnIII-2 and FnIII-2′ and toward the membrane. The arrows indicate the lack of contact between domain L1 and the domains FnIII-2 and FnIII-2′. Density in the vicinity of the ligand-free αCT element (on the surface of domain L1′) is shown in transparent magenta.

(C) Side view of the leg domains of the open-leg conformation of IGF-1Rzip.IGF-II as modeled into Map^{LO}. Density in the vicinity of the ligand-free αCT element (on the surface of domain L1′) is shown as transparent magenta surface and in the vicinity of the unmodeled domains FnIII-3 and FnIII-3′ as blue surface.

(Figure 7C). As far as can be discerned, the distance (~80 Å) between the respective C termini of FnIII-3 and FnIII-3′ is at the very limit of what is possible given the presence of the downstream zipper, allowing for both (1) a slight unraveling of the less well-packed N-terminal turn of each of the zipper helices (see, for example, PDB: 2ZTA [O'Shea et al., 1991]) and (2) an extended conformation for the native receptor residues 896–905 that link the zipper helices to their respective upstream FnIII-3 domains. By contrast, the density of the (L1′-CR′) + αCT module is relatively well defined within the open-leg map, with the αCT helix being visible in an apo-like conformation on the surface of domain L1′ and with protrusions in the map being identifiable with the aromatic residues Tyr688 and Phe692 (Figure 7C). No interpretable density is apparent for domains IDα and IDα′ apart from the αCT segment of IDα. We suggest that this open-leg conformation of the four domains FnIII-2, FnIII-2′, FnIII-2, and FnIII-3′ is likely artifactual (see Discussion).

DISCUSSION

Whereas the two structures presented here of IGF-II bound to IGF-1Rzip are similar in part to that published for the IGF-I-bound holoIGF-1R (Li et al., 2019), there is variation across these three structures in the relative disposition of domains FnIII-2, FnIII-2′, FnIII-3, and FnIII-3 and concomitantly in the separation of the points of receptor membrane entry. In none of these three structures do domains FnIII-3 and FnIII-3′ contact each other. Varying disposition of the FnIII-3 domains is also seen in the cryo-EM structure of the insulin-bound zippered IR ectodomain (Weis et al., 2018) compared with that of the insulin-bound, detergent-solubilized holo-IR (Uchikawa et al., 2019). Taken together, the extant ensemble of liganded IGF-1R and liganded IR structures thus suggests that the major consequence of ligand binding is a release of constraints on the membrane-proximal FnIII-3 domains rather than a directing of their obligate engagement. This conclusion is consistent with the extant biochemical data; once the constraints on the pair of FnIII-2 and pair of FnIII-3 domains are released, receptor activation would then be effected by *trans* association of the transmembrane and/or the cytoplasmic domains of the receptor (Kavran et al., 2014).

Nevertheless, the existence of the open-leg conformation for the IGF-II-bound ectodomain (Figures 4B and 7B) is unanticipated, as such a "wide" open-leg conformation has not been detected previously in the cryo-EM studies of insulin-bound holo-IR, insulin-bound zipper IR ectodomain, or IGF-I-bound holoIGF-1R. One possibility is that the leg conformation of structure is an artifact of the zipper attachment, which might limit the mobility of the IDα and IDα′ segments and lead—upon ligand binding—to their entrapment between domain L1′ and the respective domains FnIII-2 and FnIII-2′. One issue that has also to date been overlooked is the presence of an additional cysteine (Cys662) in the IGF-1R IDα domain, which is without counterpart in IR. Cys662 lies six residues N-terminal to the conserved cysteine triplet at residues Cys669, Cys670, and Cys 672. Mass spectroscopy analysis (see STAR Methods and Figure S6) indicates that Cys662 forms a disulfide bond with its counterpart Cys662′ in IDα′. This disulfide will add an additional constraint to the IDα segments of IGF-1R and as such may contribute to reduced mobility of these segments upon ligand binding to the zippered ectodomain. The extra disulfide may also explain why the receptor legs are closer together in apo-IGF-1R (Xu et al., 2018) than in apo-IR ectodomain (McKern et al., 2006; Croll et al., 2016) (~63 Å versus ~120 Å, respectively).

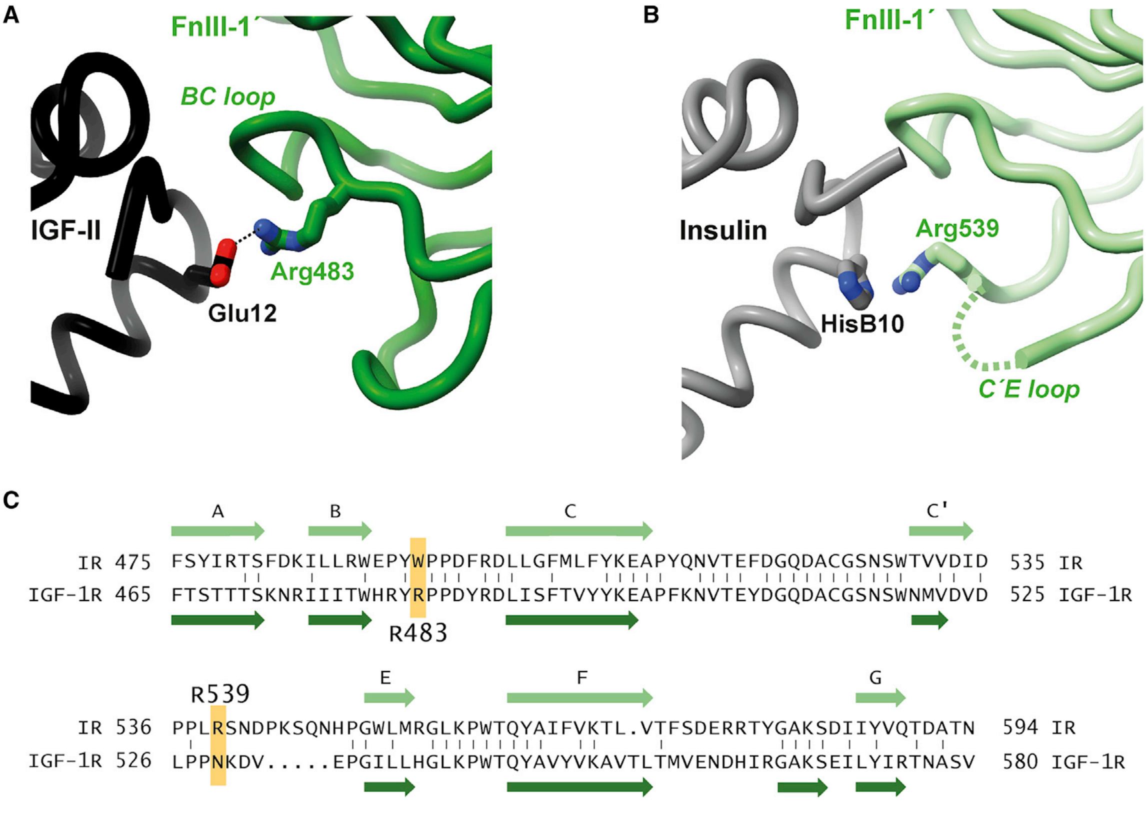

Figure 8. Interaction of IGF-II Glu12 and hIns HisB10 with the Respective FnIII-1′ Domains of IGF-1R and IR

(A) Interaction of IGF-II (black) and domain FnIII-1’ (green) as observed in the open-leg structure of IGF-II-bound IGF-1Rzip, showing the interaction of IGF-II Glu12 with IGF-1R Arg483, which lies within the BC loop of domain FnIII-1’.

(B) Juxtaposition of hIns residue HisB10 and IR residue Arg539 as observed in the structure IRΔβzip.Ins.Fv. IR Arg539 lies within the C’E loop of IR domain FnIII-1′. Replacement of insulin residue HisB10 by an aspartate residue (as in the high-affinity X10 analog) will lead to a likely salt bridge between the aspartate residue and IR Arg539. The dashed green line indicates residues between strand C′ and E that are disordered within structure IRΔβzip.Ins.Fv.

(C) Sequence alignment of domains FnIII-1′ of IR and IGF-1R, showing the non-conservation of IGF-1R Arg483 (tryptophan in IR) and non-conservation of IR Arg539 (asparagine in IGF-1R). The location of the canonical β strands A, B, C, C′, E, F, and G of the FnIII-1 domains are indicated by large arrows and labeled accordingly.

Here, only a single IGF-II molecule is seen bound to the homodimeric receptor ectodomain. However, the sample was prepared at a maximal stoichiometric ratio of 1.5 IGF-II molecules per receptor homodimer (allowing for IGF-II loss upon sample concentration; see STAR Methods); hence, at most 50% of the receptor particles could theoretically have displayed two IGF-II molecules bound. Thus, whereas we find no evidence of 3D classes reminiscent of either the two-insulin-bound, T-shaped IR ectodomain structure reported by Scapin et al. (2018), the four-insulin-bound T-shaped IR ectodomain structure reported by Gutmann et al. (2020), or the four-insulin-bound T-shaped holo-IR structure reported by Uchikawa et al. (2019), we cannot exclude the possibility of such a class arising had our ligand-to-receptor stoichiometric ratio here been higher. However, in the holoIGF-1R.IGF-I structure reported by Li et al. (2019), the stoichiometric ratio of IGF-I to holoreceptor homodimer in the sample was 2:1, yet their structure also displayed a one-to-one stoichiometry despite a high sample concentration (5 mg mL^{-1}). We suggest that these differences possibly reflect a fundamental difference between the isolated ectodomains of IGF-1R and IR: the former displays negative cooperativity of ligand binding (Surinya et al., 2008) whereas the latter does not (Markussen et al., 1991).

A major difference in the structures of the IGF-1R ectodomain-bound IGF-I and the IGF-1R ectodomain-bound IGF-II occurs in the respective growth factor C domains. In holoIGF-1R.IGF-I, IGF-I residue Tyr31, which lies near the N terminus of the C domain (Figure 1B), engages a hydrophobic pocket formed by IGF-1R L1 domain residue Pro5 and CR domain residues Phe241, Phe251, Ile255, and Pro256 (Li et al., 2019). IGF-I Tyr31 is without aromatic counterpart IGF-II, and the equivalent segments to the above of the IGF-II C domain and IGF-1R domain CR appear disordered in our structure. Instead, the IGF-II C domain appears to be stabilized at its C-terminal end by self-interactions, interactions with the N-terminal region of the IGF-II A domain, and interactions with receptor domains L1 and L2. By contrast, in holoIGF-1R.IGF-I, the C-terminal segment of IGF-I C domain appears to lack any stabilizing interactions with either the growth factor or the receptor; indeed, IGF-I residues 38–40 are unmodeled in holoIGF-1R.IGF-I (Li et al., 2019). We note that lengthening of the IGF-II C domain—by insertion of elements of the IGF-I C

domain—increases the affinity of IGF-II for IGF-1R (Henderson et al., 2015; Hexnerová et al., 2016).

As in holoIGF-1R.IGF-I, the interaction of the growth factor here with receptor domain FnIII-1′ is sparse, involving here only IGF-II B-domain residues Glu6, Thr7, Cys9, Glu12, and A-domain residues Cys47 and Phe48. Only Glu12 lies within the set of four residues (Glu12, Phe19, Leu53, and Glu57) previously identified as forming IGF-II's second receptor-binding surface (Alvino et al., 2009). We speculate that the remaining three residues (Phe19, Leu53 and Glu57) of this set of four may be involved in a transient engagement of a separate site of the receptor as part of the ligand's induced fit to the tandem (L1-CR) + αCT′ element (Xu et al., 2018). Such a site could correspond to that identified for insulin on the respective lateral surfaces of domains FnIII-1 and FnIII-1′ in cryo-EM studies of insulin-saturated IR (Uchikawa et al., 2019) and insulin-saturated IR ectodomain (Gutmann et al., 2020).

Our structure also provides some insight into the binding of mitogenic, high-affinity insulin X10 analog to IGF-1R. Insulin X10 has an aspartate substitution at residue HisB10 and has a 3- to 5-fold higher affinity for both IR and IGF-1R compared with native human insulin (Kurtzhals et al., 2000; Schwartz et al., 1987; Slieker et al., 1997). Insulin HisB10 is equivalent to IGF-II residue Glu12 and to IGF-I residue Glu9 (Figure 1B). However, the likely salt bridge observed here between the side chain of IGF-II Glu12 and IGF-1R Arg483′ (Figure 8A; conserved in holoIGF-1R.IGF-I) does not map to that predicted to occur in insulin X10 engagement with IR—namely, a salt bridge between insulin X10 AspB10 and IR Arg539′ (Figure 8B) (Weis et al., 2018). Neither IGF-1R Arg483′ nor IR Arg539′ is conserved in the alternate receptor: IGF-1R Arg483′ (= IR Trp493′) lies in the canonical BC loop of domain FnIII-1′, whereas IR Arg539′ (= IGF-1R Asn529′) lies in the canonical C'E loop of domain FnIII-1′ (Figure 8C). We suggest, therefore, that insulin X10 AspB10 exchanges its engagement site from IR Arg539′ to the non-equivalent IGF-1R Arg483′ upon binding IGF-1R. Inspection of our structure suggests that formation of an insulin X10 AspB10 to IGF-1R Arg483′ salt bridge is possible, but requires a more extended rotameric conformation for IGF-1R Arg483′ than is modeled in our IGF-II-complex IGF-1R structure in order to engage the shorter aspartate side chain.

In summary, our structure of the IGF-II-bound IGF-1R ectodomain provides insight into the way in which signaling is effected in the IGF-1R/IR subfamily of receptor tyrosine kinases and also into the structural source of the varying affinity of IGF-I and IGF-II for IGF-1R. The "resolution revolution" in cryo-EM (Kühlbrandt, 2014) has proved to be remarkably enabling in this regard, with an ensemble of cryo-EM structures of the subfamily appearing over the past two years, compared with the almost decade-long gap between structures when the sole tool available was X-ray crystallography. A major challenge, however, remains—namely, elucidating the structural pathway by which the ligand (IGF-I, IGF-II) enters the partially occluded primary binding site formed by IGF-1R domain L1 and the receptor αCT′ segment. Some insight into this issue has recently been gained by the discovery of a further insulin-binding site on the surface of the FnIII-1 domains of IR (Uchikawa et al., 2019), but the existence of an equivalent site has yet to be confirmed for IGF-1R.

STAR★METHODS

Detailed methods are provided in the online version of this paper and include the following:

- KEY RESOURCES TABLE
- RESOURCE AVAILABILITY
  - Lead Contact
  - Materials Availability
  - Data and Code Availability
- EXPERIMENTAL MODEL AND SUBJECT DETAILS
  - CHO-K1 Cells
  - BALB/c3T3 Overexpressing IGF-IR (P6) Cells
- METHOD DETAILS
  - Cloning and Production of IGF-1Rzip
  - Purification of IGF-1Rzip
  - Europium Labelling of Peptides
  - Receptor Competition Binding Assay
  - Preparation of IGF-II-Complexed IGF-1Rzip
  - CryoEM Grid Preparation
  - CryoEM Data Collection
  - Three-Dimensional Single-Particle Reconstruction
  - Model Building
  - Identification of Disulfide Links Using Mass Spectrometry (MS)
- QUANTITATION AND STATISTICAL ANALYSIS

SUPPLEMENTAL INFORMATION

Supplemental Information can be found online at https://doi.org/10.1016/j.str.2020.05.002.

ACKNOWLEDGMENTS

The authors acknowledge use of facilities within the Monash Ramaciotti Cryo EM platform. M.C.L. acknowledges financial support from the Australian National Health and Medical Research Council (NHMRC) project grant APP1128553; his institute receives Victorian State Government Operational Infrastructure support and funding from the Australian NHMRC Independent Research Institutes Infrastructure support scheme. T.I.C. is supported by the Wellcome Trust Principal Research Fellowship of Prof. Randy Read (grant number 209407/Z/17/Z). The authors thank Prof. Ken Siddle (University of Cambridge, England) for providing the hybridomas expressing mAb 24-60, and Dr. Oliver Clarke (Columbia University, USA) for advice regarding cryo-EM software. Molecular graphics and analyses were performed with UCSF ChimeraX, developed by the Resource for Biocomputing, Visualization, and Informatics at the University of California, San Francisco, with support from National Institutes of Health R01-GM129325 and the Office of Cyber Infrastructure and Computational Biology, National Institute of Allergy and Infectious Diseases. This paper is dedicated to our late colleague and mentor, Dr. Colin Ward, who initiated our structural studies of IGF-1R.

AUTHOR CONTRIBUTIONS

M.B.M. performed molecular and cell biology. Y.X. and M.B.M. performed protein purification and sample preparation. H.V. and Y.X. performed cryo-EM experiments. C.A.D. performed assays. Y.X. and N.S.K. performed cryo-EM three-dimensional reconstruction. T.I.C., Y.X., N.S.K., and M.C.L. performed model building and refinement. J.J.S. and A.I.W. performed mass spectroscopy experiments. M.C.L. designed the project. M.C.L. and B.E.F. analyzed results and wrote the manuscript. All authors read and commented on the final manuscript.

DECLARATION OF INTERESTS

M.C.L.'s laboratory has a funded agreement with Eli Lilly and Company to conduct research not connected to this publication.

Received: February 27, 2020
Revised: April 23, 2020
Accepted: May 5, 2020
Published: May 26, 2020

REFERENCES

Adams, T.E., Epa, V.C., Garrett, T.P., and Ward, C.W. (2000). Structure and function of the type 1 insulin-like growth factor receptor. Cell. Mol. Life Sci. *57*, 1050–1093.

Afonine, P.V., Poon, B.K., Read, R.J., Sobolev, O.V., Terwilliger, T.C., Urzhumtsev, A., and Adams, P.D. (2018). Real-space refinement in PHENIX for cryo-EM and crystallography. Acta Crystallogr. D Biol. Crystallogr. *74*, 531–544.

Alvino, C.L., McNeil, K.A., Ong, S.C., Delaine, C., Booker, G.W., Wallace, J.C., Whittaker, J., and Forbes, B.E. (2009). A novel approach to identify two distinct receptor binding surfaces of insulin-like growth factor II. J. Biol. Chem. *284*, 7656–7664.

Barron, J. (2019). A general and adaptive robust loss function. Proc. IEEE Conf. Comput. Vis. Pattern Recognit. 4331–4339.

Baxter, R.C. (2014). IGF binding proteins in cancer: mechanistic and clinical insights. Nat. Rev. Cancer *14*, 329–341.

Belfiore, A., Malaguarnera, R., Vella, V., Lawrence, M.C., Sciacca, L., Frasca, F., Morrione, A., and Vigneri, R. (2017). Insulin receptor isoforms in physiology and disease: an updated view. Endocr. Rev. *38*, 379–431.

Christoffersen, C.T., Bornfeldt, K.E., Rotella, C.M., Gonzales, N., Vissing, H., Shymko, R.M., ten Hoeve, J., Groffen, J., Heisterkamp, N., and De Meyts, P. (1994). Negative cooperativity in the insulin-like growth factor-I receptor and a chimeric IGF-I/insulin receptor. Endocrinology *135*, 472–475.

Cox, J., and Mann, M. (2008). MaxQuant enables high peptide identification rates, individualized p.p.b.-range mass accuracies and proteome-wide protein quantification. Nat. Biotechnol. *26*, 1367–1372.

Croll, T. (2018). ISOLDE: a physically realistic environment for model building into low-resolution electron-density maps. Acta Crystallogr. D Biol. Crystallogr. *74*, 519–530.

Croll, T.I., Smith, B.J., Margetts, M.B., Whittaker, J., Weiss, M.A., Ward, C.W., and Lawrence, M.C. (2016). Higher-resolution structure of the human insulin receptor ectodomain: multi-modal inclusion of the insert domain. Structure *24*, 469–476.

Denley, A., Bonython, E.R., Booker, G.W., Cosgrove, L.J., Forbes, B.E., Ward, C.W., and Wallace, J.C. (2004). Structural determinants for high-affinity binding of insulin-like growth factor II to insulin receptor (IR)-A, the exon 11 minus isoform of the IR. Mol. Endocrinol. *18*, 2502–2512.

Denley, A., Cosgrove, L.J., Booker, G.W., Wallace, J.C., and Forbes, B.E. (2005). Molecular interactions of the IGF system. Cytokine Growth Factor Rev. *16*, 421–439.

Dyer, A.H., Vahdatpour, C., Sanfeliu, A., and Tropea, D. (2016). The role of insulin like growth factor 1 (IGF-1) in brain development, maturation and neuroplasticity. Neuroscience *325*, 89–99.

El-Shewy, H.M., and Luttrell, L.M. (2009). Insulin-like growth factor-2/mannose-6 phosphate receptors. Vitam. Horm. *80*, 667–697.

Emsley, P., Lohkamp, B., Scott, W.G., and Cowtan, K. (2010). Features and development of Coot. Acta Crystallogr. D Biol. Crystallogr. *66*, 486–501.

Francis, G.L., Aplin, S.E., Milner, S.J., McNeil, K.A., Ballard, F.J., and Wallace, J.C. (1993). Insulin-like growth factor (IGF)-II binding to IGF-binding proteins and IGF receptors is modified by deletion of the N-terminal hexapeptide or substitution of arginine for glutamate-6 in IGF-II. Biochem. J. *293*, 713–719.

Goddard, T.D., Huang, C.C., Meng, E.C., Pettersen, E.F., Couch, G.S., Morris, J.H., and Ferrin, T.E. (2018). UCSF ChimeraX: meeting modern challenges in visualization and analysis. Protein Sci. *27*, 14–25.

Götze, M., Pettelkau, J., Schaks, S., Bosse, K., Ihling, C.H., Krauth, F., Fritzsche, R., Kühn, U., and Sinz, A. (2012). StavroX—a software for analyzing crosslinked products in protein interaction studies. J. Am. Soc. Mass Spectrom. *23*, 76–87.

Gutmann, T., Schäfer, I.B., Poojari, C., Brankatschk, B., Vattulainen, I., Strauss, M., and Coskun, Ü. (2020). Cryo-EM structure of the complete and ligand-saturated insulin receptor ectodomain. J. Cell Biol. *219*, e201907210.

Henderson, S.T., Brierley, G.V., Surinya, K.H., Priebe, I.K., Catcheside, D.E., Wallace, J.C., Forbes, B.E., and Cosgrove, L.J. (2015). Delineation of the IGF-II C domain elements involved in binding and activation of the IR-A, IR-B and IGF-IR. Growth Horm. IGF Res. *25*, 20–27.

Hexnerová, R., Křížková, K., Fábry, M., Sieglová, I., Kedrová, K., Collinsová, M., Ullrichová, P., Srb, P., Williams, C., Crump, M.P., et al. (2016). Probing receptor specificity by sampling the conformational space of the insulin-like growth factor II C-domain. J. Biol. Chem. *291*, 21234–21245.

Holly, J.M.P., Biernacka, K., and Perks, C.M. (2019). The neglected insulin: IGF-II, a metabolic regulator with implications for diabetes, obesity, and cancer. Cells *8*, 1207.

Hoogenboom, H.R., Griffiths, A.D., Johnson, K.S., Chiswell, D.J., Hudson, P., and Winter, G. (1991). Multi-subunit proteins on the surface of filamentous phage: methodologies for displaying antibody (Fab) heavy and light chains. Nucleic Acids Res. *19*, 4133–4137.

Hoyne, P.A., Cosgrove, L.J., McKern, N.M., Bentley, J.D., Ivancic, N., Elleman, T.C., and Ward, C.W. (2000). High affinity insulin binding by soluble insulin receptor extracellular domain fused to a leucine zipper. FEBS Lett. *479*, 15–18.

Kavran, J.M., McCabe, J.M., Byrne, P.O., Connacher, M.K., Wang, Z., Ramek, A., Sarabipour, S., Shan, Y., Shaw, D.E., Hristova, K., et al. (2014). How IGF-1 activates its receptor. Elife *3*, e03772.

King, R., Wells, J.R., Krieg, P., Snoswell, M., Brazier, J., Bagley, C.J., Wallace, J.C., Ballard, F.J., Ross, M., and Francis, G.L. (1992). Production and characterization of recombinant insulin-like growth factor-I (IGF-I) and potent analogues of IGF-I, with Gly or Arg substituted for Glu3, following their expression in *Escherichia coli* as fusion proteins. J. Mol. Endocrinol. *8*, 29–41.

Kühlbrandt, W. (2014). Biochemistry. The resolution revolution. Science *343*, 1443–1444.

Kurtzhals, P., Schäffer, L., Sørensen, A., Kristensen, C., Jonassen, I., Schmid, C., and Trüb, T. (2000). Correlations of receptor binding and metabolic and mitogenic potencies of insulin analogs designed for clinical use. Diabetes *49*, 999–1005.

Laviola, L., Natalicchio, A., and Giorgino, F. (2007). The IGF-I signaling pathway. Curr. Pharm. Des. *13*, 663–669.

Lemmon, M.A., and Schlessinger, J. (2010). Cell signaling by receptor tyrosine kinases. Cell *141*, 1117–1134.

Li, J., Choi, E., Yu, H., and Bai, X.C. (2019). Structural basis of the activation of type 1 insulin-like growth factor receptor. Nat. Commun. *10*, 4567.

Macháčková, K., Mlčochová, K., Potalitsyn, P., Hanková, K., Socha, O., Buděšínský, M., Muždalo, A., Lepšík, M., Černeková, M., Radosavljević, J., et al. (2019). Mutations at hypothetical binding site 2 in insulin and insulin-like growth factors 1 and 2 result in receptor- and hormone-specific responses. J. Biol. Chem. *294*, 17371–17382.

Markussen, J., Halstrøm, J., Wiberg, F.C., and Schäffer, L. (1991). Immobilized insulin for high capacity affinity chromatography of insulin receptors. J. Biol. Chem. *266*, 18814–18818.

McKern, N.M., Lawrence, M.C., Streltsov, V.A., Lou, M.Z., Adams, T.E., Lovrecz, G.O., Elleman, T.C., Richards, K.M., Bentley, J.D., Pilling, P.A., et al. (2006). Structure of the insulin receptor ectodomain reveals a folded-over conformation. Nature *443*, 218–221.

McKern, N.M., Lou, M., Frenkel, M.J., Verkuylen, A., Bentley, J.D., Lovrecz, G.O., Ivancic, N., Elleman, T.C., Garrett, T.P., Cosgrove, L.J., and Ward, C.W. (1997). Crystallization of the first three domains of the human insulin-like growth factor-1 receptor. Protein Sci. *6*, 2663–2666.

Nakane, T., Kimanius, D., Lindahl, E., and Scheres, S.H. (2018). Characterisation of molecular motions in cryo-EM single-particle data by multi-body refinement in RELION. Elife *7*, e36861.

O'Shea, E.K., Klemm, J.D., Kim, P.S., and Alber, T. (1991). X-ray structure of the GCN4 leucine zipper, a two-stranded, parallel coiled coil. Science *254*, 539–544.

Pandini, G., Frasca, F., Mineo, R., Sciacca, L., Vigneri, R., and Belfiore, A. (2002). Insulin/insulin-like growth factor I hybrid receptors have different biological characteristics depending on the insulin receptor isoform involved. J. Biol. Chem. *277*, 39684–39695.

Pettersen, E.F., Goddard, T.D., Huang, C.C., Couch, G.S., Greenblatt, D.M., Meng, E.C., and Ferrin, T.E. (2004). UCSF Chimera—a visualization system for exploratory research and analysis. J. Comput. Chem. *25*, 1605–1612.

Pietrzkowski, Z., Lammers, R., Carpenter, G., Soderquist, A.M., Limardo, M., Phillips, P.D., Ullrich, A., and Baserga, R. (1992). Constitutive expression of insulin-like growth factor 1 and insulin-like growth factor 1 receptor abrogates all requirements for exogenous growth factors. Cell Growth Differ *3*, 199–205.

Punjani, A., Rubinstein, J.L., Fleet, D.J., and Brubaker, M.A. (2017). cryoSPARC: algorithms for rapid unsupervised cryo-EM structure determination. Nat. Methods *14*, 290–296.

Riedemann, J., and Macaulay, V.M. (2006). IGF1R signalling and its inhibition. Endocr. Relat. Cancer *13* (*Suppl 1*), S33–S43.

Scapin, G., Dandey, V.P., Zhang, Z., Prosise, W., Hruza, A., Kelly, T., Mayhood, T., Strickland, C., Potter, C.S., and Carragher, B. (2018). Structure of the insulin receptor–insulin complex by single-particle cryo-EM analysis. Nature *556*, 122–125.

Schwartz, G.P., Burke, G.T., and Katsoyannis, P.G. (1987). A superactive insulin: [B10-aspartic acid]insulin(human). Proc. Natl. Acad. Sci. USA *84*, 6408–6411.

Slieker, L.J., Brooke, G.S., DiMarchi, R.D., Flora, D.B., Green, L.K., Hoffmann, J.A., Long, H.B., Fan, L., Shields, J.E., Sundell, K.L., et al. (1997). Modifications in the B10 and B26-30 regions of the B chain of human insulin alter affinity for the human IGF-I receptor more than for the insulin receptor. Diabetologia *40*, S54–S61.

Smith, B.J., Huang, K., Kong, G., Chan, S.J., Nakagawa, S., Menting, J.G., Hu, S.-Q., Whittaker, J., Steiner, D.F., Katsoyannis, P.G., et al. (2010). Structural resolution of a tandem hormone-binding element in the insulin receptor and its implications for design of peptide agonists. Proc. Natl. Acad. Sci. U S A *107*, 6771–6776.

Soos, M.A., Field, C.E., Lammers, R., Ullrich, A., Zhang, B., Roth, R.A., Andersen, A.S., Kjeldsen, T., and Siddle, K. (1992). A panel of monoclonal antibodies for the type I insulin-like growth factor receptor. Epitope mapping, effects on ligand binding, and biological activity. J. Biol. Chem. *267*, 12955–12963.

Surinya, K.H., Forbes, B.E., Occhiodoro, F., Booker, G.W., Francis, G.L., Siddle, K., Wallace, J.C., and Cosgrove, L.J. (2008). An investigation of the ligand binding properties and negative cooperativity of soluble insulin-like growth factor receptors. J. Biol. Chem. *283*, 5355–5363.

Tao, Y., Pinzi, V., Bourhis, J., and Deutsch, E. (2007). Mechanisms of disease: signaling of the insulin-like growth factor 1 receptor pathway-therapeutic perspectives in cancer. Nat. Clin. Pract. Oncol. *4*, 591–602.

Torres, A.M., Forbes, B.E., Aplin, S.E., Wallace, J.C., Francis, G.L., and Norton, R.S. (1995). Solution structure of human insulin-like growth factor II. Relationship to receptor and binding protein interactions. J. Mol. Biol. *248*, 385–401.

Uchikawa, E., Choi, E., Shang, G., Yu, H., and Bai, X.-c. (2019). Activation mechanism of the insulin receptor revealed by cryo-EM structure of the fully liganded receptor-ligand complex. Elife *8*, e48630.

Ullrich, A., Gray, A., Tam, A.W., Yang-Feng, T., Tsubokawa, M., Collins, C., Henzel, W., Le Bon, T., Kathuria, S., Chen, E., et al. (1986). Insulin-like growth factor I receptor primary structure: comparison with insulin receptor suggests structural determinants that define functional specificity. EMBO J. *5*, 2503–2512.

Weis, F., Menting, J.G., Margetts, M.B., Chan, S.J., Xu, Y., Tennagels, N., Wohlfart, P., Langer, T., Müller, C.W., Dreyer, M.K., and Lawrence, M.C. (2018). The signalling conformation of the insulin receptor ectodomain. Nat. Commun. *9*, 4420.

Whitten, A.E., Smith, B.J., Menting, J.G., Margetts, M.B., McKern, N.M., Lovrecz, G.O., Adams, T.E., Richards, K., Bentley, J.D., Trewhella, J., et al. (2009). Solution structure of ectodomains of the insulin receptor family: the ectodomain of the type 1 insulin-like growth factor receptor displays asymmetry of ligand binding accompanied by limited conformational change. J. Mol. Biol. *394*, 878–892.

Williams, C.J., Headd, J.J., Moriarty, N.W., Prisant, M.G., Videau, L.L., Deis, L.N., Verma, V., Keedy, D.A., Hintze, B.J., Chen, V.B., et al. (2018). MolProbity: more and better reference data for improved all-atom structure validation. Protein Sci. *27*, 293–315.

Wisniewski, J.R., Zougman, A., and Mann, M. (2009). Combination of FASP and StageTip-based fractionation allows in-depth analysis of the hippocampal membrane proteome. J. Proteome Res. *8*, 5674–5678.

Xu, Y., Kong, G.K.W., Menting, J.G., Margetts, M.B., Delaine, C.A., Jenkin, L.M., Kiselyov, V.V., De Meyts, P., Forbes, B.E., and Lawrence, M.C. (2018). How ligand binds to the type 1 insulin-like growth factor receptor. Nat. Commun. *9*, 821.

STAR★METHODS

KEY RESOURCES TABLE

REAGENT or RESOURCE	SOURCE	IDENTIFIER
Antibodies		
Mouse monoclonal antibody (anti-human IGF-1R)	(Soos et al., 1992)	24-60
Mouse monoclonal antibody (anti-human IGF-1R)	(Soos et al., 1992)	24-31
Mouse monoclonal antibody 9E10 (anti-c-myc)	CSIRO Laboratories, Parkville, Australia	ATCC CRL1729
IRDye 800CW Goat anti-mouse IgG	Millennium Science, Australia	Cat# 926-32210
Chemicals, Peptides, and Recombinant Proteins		
9E10 c-myc peptide, sequence EQKLISEEDL (>75% purity)	Genscript	N/A
Receptor grade IGF-II	GroPep	Cat# FM001
IGF-I	(King et al., 1992)	N/A
IGF-II	(Francis et al., 1993)	N/A
Eu-IGF-I	(Denley et al., 2004)	N/A
Eu-IGF-II	(Denley et al., 2004)	N/A
Fetal calf serum	Scientifix	Cat# FBSFR-62147A
G418	ThermoFisher Scientific	Cat# 10131035
Eu-N1-ITC chelate	Perkin Elmer	Cat# 1244-302
Fetal Bovine Serum, dialyzed	ThermoFisher Scientific	Cat# 30067344
X-tremeGENE 9 transfection agent	Roche	Cat# 06 365 787 001
DMEM with Glucose, without L-Glutamine	Lonza	Cat# 12-614F
GS Supplement	Merck	Cat# GSS-1016-C
Valproic acid	Sigma-Aldrich / Merck	Cat# P4543
Trypsin Gold	Promega	Cat# V5280
Critical Commercial Assays		
DELFIA Eu-labeling kit	PerkinElmer	Cat# 1244-302
DELFIA Enhancement Solution	Perkin Elmer	Cat# 1244-104
Deposited Data		
Gene sequence of *Homo sapiens* IGF-1R	Genome Reference Consortium	UniProt: P08069
Gene sequence of *Saccharomyces cerevisiae* GCN4	Saccharomyces Genome Database	UniProt: P03069
CryoEM structure of IGF-I-bound holo IGF-1R	(Li et al., 2019)	PDB: 6PYH
Crystal structure of GCN4 leucine zipper	(O'Shea et al., 1991)	PDB: 2ZTA
Crystal structure of apo IGF-1R ectodomain	(Xu et al., 2018)	PDB: 5U8R
Crystal structure of IGF-I-bound IGF-1R ectodomain	(Xu et al., 2018)	PDB: 5U8Q
Model: IGF-II-bound IGF-1Rzip, head region, closed-leg	This study	PDB: 6VWI
Model: IGF-II-bound IGF-1Rzip, leg region, open-leg	This study	PDB: 6VWH
Model: IGF-II-bound IGF-1Rzip, head region, open-leg	This study	PDB: 6VWG
Model: IGF-II-bound IGF-1Rzip, leg region, closed-leg	This study	PDB: 6VWJ
Map: IGF-II-bound IGF-1Rzip, head region, open-leg	This study	EMD-21417
Map: IGF-II-bound IGF-1Rzip, head region, closed-leg	This study	EMD-21415
Map: IGF-II-bound IGF-1Rzip, leg region, open-leg	This study	EMD-21416
Map: IGF-II-bound IGF-1Rzip, leg region, closed-leg	This study	EMD-21418
Experimental Models: Cell Lines		
BALB/c3T3 cells overexpressing IGF-IR (sex: unknown)	(Pietrzkowski et al., 1992)	P6
Chinese Hamster Ovary (CHO) K1 cells (sex: female)	ATCC	ATCC CCL-61

(*Continued on next page*)

Continued

REAGENT or RESOURCE	SOURCE	IDENTIFIER
Recombinant DNA		
IGF-1Rzip, custom synthesis	Genscript	N/A
pEE14 vector	Lonza	N/A
Software and Algorithms		
RELION v3.0.5	(Nakane et al., 2018)	https://www3.mrc-lmb.cam.ac.uk/relion
CryoSPARC v2.11	(Punjani et al., 2017)	https://cryosparc.com/
ISOLDE v1.03b	(Croll, 2018)	https://isolde.cimr.cam.ac.uk/
Phenix v1.16-3549-000	(Afonine et al., 2018)	https://www.phenix-online.org/
Coot v0.8.9.1	(Emsley et al., 2010)	https://www2.mrc-lmb.cam.ac.uk/personal/pemsley/coot/
Chimera v1.11.2	(Pettersen et al., 2004)	https://www.cgl.ucsf.edu/chimera/
ChimeraX v0.91	(Goddard et al., 2018)	https://www.cgl.ucsf.edu/chimerax/
Graphpad Prism v8.0.2	Graphpad Software	https://www.graphpad.com/scientific-software/prism/
MaxQuant V1.6.7.0	(Cox and Mann, 2008)	https://www.maxquant.org/
StavroX V3.6.6.5	(Götze et al., 2012)	http://www.stavrox.com/
Exactive V2.1 build 1502	ThermoFisher Scientific	N/A
APL to MGF converter software	https://www.wehi.edu.au/people/andrew-webb/1298/apl-mgf-converter	N/A
Xcalibur 3.0	ThermoFisher Scientific	N/A
Other		
Mini-Leak low divinylsulphone-activated resin	Kem-en-Tec	Cat# 1011 H
Sepharose CL-4B resin	GE Healthcare Lifesciences	Cat# 17015001
Sephadex-G75	GE Healthcare / Cytiva	Cat# 17005001
Pellicon 3 0.11 m^2 10 kDa Ultracel concentrator	Merck-Millipore	Cat# P3C010C01
Bottle filter, 0.2 μm (Nalgene)	ThermoFisher Scientific	Cat# 567-0020
Superdex 200 Increase 10/300 GL	GE Healthcare Lifesciences	Cat# 28990944
0.5 mL 10 kDa Amicon Ultra concentrator	Sigma-Aldrich	Cat# UFC501008
UltrAuFoil R1.2/1.3 300-mesh grids	Quantifoil	N/A
Pelco easiGlow	Ted Pella	Cat# 91000S-230
Vitrobot mark IV	ThermoFisher Scientific	N/A
Aurora packed emitter column	IonOpticks	Cat# AUR2-25075C18A

RESOURCE AVAILABILITY

Lead Contact

Further information and requests for resources and reagents should be directed to and will be fulfilled by the Lead Contact, Michael Lawrence (lawrence@wehi.edu.au).

Materials Availability

There are restrictions to the availability of the vector and stable cell lines associated with the IGF-1Rzip construct due to the pEE14 vector being subject to a Research Agreement with Lonza.

Data and Code Availability

Map^{HO}, Map^{LO}, Map^{HC} and Map^{LC} along with their associated atomic models have been deposited in the Electron Microscopy Data Bank and Protein Data Bank (EMDB entries EMD-21417, EMD-21416, EMD-21415 and EMD-21418, and PDB entries 6VWG, 6VWH, 6VWI and 6VWJ, respectively).

EXPERIMENTAL MODEL AND SUBJECT DETAILS

CHO-K1 Cells

CHO-K1 cells (ATCC CCL-61) stably transfected with pEE14 plasmid containing the IGF-1Rzip gene were cultured at 37°C in DMEM (high glucose) media containing 25 μM methionine sulfoxide, 1× GS supplement and 10% dialysed fetal bovine serum.

BALB/c3T3 Overexpressing IGF-IR (P6) Cells

BALB/c3T3 overexpressing IGF-IR (P6) cells were cultured in DMEM, 10% fetal calf serum, 1% penicillin/streptomycin, G418 (0.5 mM), at 37°C, 5% CO_2. The P6 cells were a gift from Dr Renato Baserga (Pietrzkowski et al., 1992) and were validated for over-expression of IGF-1R by FACS analysis.

METHOD DETAILS

Cloning and Production of IGF-1Rzip

A synthetic gene encoding IGF-1Rzip (comprising, in order, a 399-nucleotide stretch of pre-signal native sequence followed by a gene encoding the native signal peptide, residues 1-905 of IGF-1R (UniProt entry P08069-1), a 33-residue GCN4 zipper sequence RMKQLEDKVEELLSKNYHLENEVARLKKLVGER (UniProt entry P03069), a three-serine spacer and the c-myc tag sequence EQKLI-SEEDLN) was cloned into the Hind III / Xba1 sites (Genscript; Piscataway, New Jersey) of the pEE14 mammalian expression vector (Lonza; Basel, Switzerland) for stable expression of the protein in CHO-K1 cells. Cells were transfected with complexes of plasmid DNA and X-tremeGENE 9 transfection agent (Roche; Basel, Switzerland) then later selected with 25 μM methionine sulfoxide (Merck; Darmstadt, Germany) in DMEM (High Glucose) media (Lonza) containing 1× GS supplement (Merck) and 10% dialysed fetal bovine serum (ThermoFisher Scientific / Life Technologies; Waltham, Massachusetts). Cells were plated in 96-well plates using limiting dilution and colonies were allowed to form over several weeks. Secretion of target protein from colonies was detected *via* Western blot using mAb 24-60 (Soos et al., 1992) (hybridomas expressing mAb 24-60 were a gift of Professor Ken Siddle, Cambridge, UK). Dozens of colonies were amplified into twelve-well trays and later tissue culture flasks and monitored for expression *via* Western blot (as above). Several of the best-expressing clones were then further screened by seeding cells at exactly the same densities in six-well trays and individually monitored for expression over time. The single best-expressing clone was then selected to enter roller bottle scale-up. Cells were seeded in roller bottles and allowed to grow for 21 days with the addition of 2.5 mM valproic acid (Sigma-Aldrich/ Merck; Darmstadt, Germany) at day 14. Finally, the conditioned media were decanted from the roller bottles and filtered for purification through a Nalgene 0.2 μm bottle filter (ThermoFisher Scientific).

Purification of IGF-1Rzip

IGF-1Rzip was purified from a single 5-L batch of conditioned medium to which was added PMSF (1:1000 dilution of 100 mM PMSF/ propan-2-ol; Merck) and sodium azide (0.02 %; Sigma-Aldrich). Sample volume reduction, for ease of purification, was achieved by cycling the conditioned medium at room temperature through a stack of two Pellicon 3 0.11 m^2 10 kDa concentrator cartridges (Merck-Millipore; Darmstadt, Germany) until the concentrate volume was 500 mL. For purification, the filtered concentrate was flowed through a 100-mL bed volume (BV), 50-cm diameter, Sepharose CL-4B guard column (GE Healthcare Lifesciences; Marlborough, Massachusetts) to remove non-specifically adsorbing material and then over a 40-mL mAb 9E10 50-cm affinity column at a flow rate of 2 $mL.min^{-1}$; the affinity matrix being prepared by coupling mAb 9E10 (CSIRO; Parkville Australia) directly to Mini-Leak Low divinylsulphone-activated agarose resin (Kem-En-Tec; Tasstrup, Denmark) (McKern et al., 1997). The flow-through was re-loaded several times onto the latter column with a final overnight bind at 4 °C. The affinity column was then washed with two column volumes (CV) of Tris-buffered saline with 0.02 % azide [24.8 mM Tris-HCl (pH 8.0), 137 mM NaCl, 2.7 mM KCl, and 0.02% sodium azide; "TBSA"] containing 0.1 mM PMSF (this wash was retained and added to the conditioned medium for a second bind) followed by a 10 CV TBSA wash which was discarded. Bound protein was eluted by recycling a 50 mL solution of c-myc peptide EQKLI-SEEDLN (0.2 $mg.mL^{-1}$ in TBSA) over the column nine times with a final chase of 50 mL fresh peptide solution. An Amicon (Merck-Millipore) stirred cell concentrator with a 30 kDa disc filter was used to concentrate 100 mL of eluate to 10 mL and finally to 1 mL in a centrifugal concentrator before a further purification step on a serial pair of Superdex™ 200 Increase 10/300 GL (GE Healthcare) size-exclusion chromatography (SEC) columns. The SEC profile (Figure S1A) displayed two peaks corresponding to the approximate respective sizes of the desired IGF-1Rzip (i.e., dimeric $(\alpha\beta)_2$) protein and an unwanted $(\text{IGF-1Rzip})_2$ (i.e., tetrameric $(\alpha\beta)_4$) protein. The IGF-1Rzip-containing fractions were then re-run twice through the same column pair to remove as far as possible any residual $(\text{IGF-1Rzip})_2$ protein without substantial loss of IRGF-1Rzip (Figures S1B and 1C). The identity of the IGF-1Rzip protein was confirmed by sodium-dodecyl-sulfate polyacrylamide gel electrophoresis (SDS-PAGE; Figure S1D), followed by Western blot analysis with mAb 24-60 (data not shown).

Europium Labelling of Peptides

Europium-labeled human IGF-I (King et al., 1992) and human IGF-II (Francis et al., 1993) were prepared as instructed by the manufacturer (DELFIA Eu-labeling kit, Perkin Elmer) (Denley et al., 2004). Peptide (0.43 mM) was incubated with 2 mM labeling reagent in a 30 μl reaction (0.1 M Na_2CO_3, pH 8.5), at 4 °C for 2 d. The reaction was terminated with 0.05 M Tris-HCl, 0.15 M NaCl (pH 7.5), and

unbound europium was removed by SEC (Sephadex 75, GE Healthcare/Cytiva) in the termination buffer. Aliquots were stored at 50 mmol.L^{-1} Tris-HCl buffered saline solution containing 0.1-0.5% purified BSA, 0.05% sodium azide.

Receptor Competition Binding Assay

BALB/c3T3 overexpressing IGF-IR (P6) cells (Pietrzkowski et al., 1992) were cultured in DMEM, 10% fetal calf serum, 1% penicillin/streptomycin, G418 (0.5 mM). IGF-IR was solubilized from P6 cells using lysis buffer [20 mM HEPES, 150 mM NaCl, 1.5 mM $MgCl_2$, 10% (v/v) glycerol, 1% (v/v) Triton X-100, 1 mM EGTA (pH 7.5)] for 1 h at 4°C and lysates were centrifuged for 10 min at 3500 rpm. Solubilized IGF-1R (100 μl) or IGF-1Rzip (0.5 μg) was used to coat each well of a white Greiner Lumitrac 600 plate previously coated with 24-31 anti-human IGF-1R antibody (Soos et al., 1992). Europium-labelled IGF-I or IGF-II (~100,000 counts) were added to wells with increasing concentrations of competitive ligand IGF-I or IGF-II and incubated for 16 h at 4°C. Wells were washed with 20 mM Tris, 150 mM NaCl, 0.05% (v/v) Tween 20 and DELFIA enhancement solution (100 μl) was added. Time-resolved fluorescence was measured with 340-nm excitation and 612-nm emission filters with a Polarstar Fluorimeter (BMG Labtech). Replicate details are as follows: IGF-I versus holo-IGF-1R, three assays with three replicates (n=9, with a single individual measurement omitted as aberrant); IGF-I versus IGF-1Rzip, three independent assays with three replicates each (n=9); IGF-II versus holoIGF-1R, two independent assays with three replicates each (n=6); IGF-II versus IGF-1Rzip, three independent assays with three replicates each (n=9, with four individual measurement omitted as aberrant) (see Figures S1E and S1F). Mean IC_{50} values were calculated with the statistical software package Prism v8.0.2 (GraphPad Software) after curve fitting with non-linear regression (one-site) model. Qualitative assessment of the difference of affinities of each ligand for the IGF-1Rzip versus holo-IGRF-1R were based on an *F* test within Prism (IGF-I: no. degrees of freedom = 164; IGF-II : no. degrees of freedom = 131).

Preparation of IGF-II-Complexed IGF-1Rzip

"Receptor grade" IGF-II (GroPep; Thebarton, Australia) was dissolved in 10 mM HCl to a concentration of 5.9 mg.mL^{-1} and then added to IGF-1Rzip (pre-prepared at a concentration of 0.19 mg.mL^{-1} in TBSA) to give a final IGF-1Rzip to IGF-II molar ratio of 1 to 1.5 (i.e., of receptor homodimer to ligand). A stock solution was then prepared by concentrating the mixture to 1.09 mg.mL^{-1} in TBSA using a 0.5 mL 10 kDa Amicon Ultra concentrator (Sigma Aldrich). Aliquots of stock solution were diluted with TBSA to 0.1 mg.mL^{-1} (nominally 0.5 μM IGF-1Rzip homodimer plus maximum 0.75 μM IGF-II) to provide sample volumes for cryoEM analysis.

CryoEM Grid Preparation

A 4 μL volume of the above stock solution was then applied to UltrAuFoil R1.2/1.3 300-mesh grids (Quantifoil Micro Tools GmbH; Großlöbichau, Germany) which were glow discharged prior to sample application in a Pelco easiGlow device (Ted Pella; Redding, California) at 15 mA for 30 s. Grids were blotted for 3 s at -3 blot-force setting in a Vitrobot mark IV (ThermoFisher Scientific; operated at 4°C and 100 % humidity) before being plunge frozen in liquid ethane.

CryoEM Data Collection

CryoEM imaging was performed using a Titan Krios (ThermoFisher Scientific) equipped with a Gatan K2 Summit™ with Quantum-GIF energy filter. Imaging was performed in nanoprobe energy filtered zero loss mode using a 20 eV slit width. A nominal magnification of 130,000 × was used which provided a calibrated specimen level pixel size of 1.06 Å. A C2 condenser aperture of 50 μm and an objective aperture of 100 μm were used and the K2 camera was operated in counting mode at a dose rate of 6 e^-/pixel/s. Each movie was collected using a 10 s exposure time fractionated into 50 sub-frames resulting in a total accumulated dose of 50 $e^-/Å^2$ per movie. The EPU software package (ThermoFisher Scientific) was used for automated data collection and a total of 4585 movies were collected using a defocus range from -0.6 μm to -1.6 μm.

Three-Dimensional Single-Particle Reconstruction

The data set of 4585 movies were gain-, motion- and dose corrected within RELION 3.0.5 (Nakane et al., 2018). The motioned-corrected images were then corrected for contrast transfer function (CTF) using CTFFIND v4.1.13, also within RELION. Images showing ice contamination, low particle number or poor CTF fit were excluded, resulting in a final set of 3963 images. 2057701 particles then were auto-picked (using templates obtained from an in-house study of IGF-I-bound to IGF-1Rzip; data not shown) and then extracted within RELION. This latter set of particles was then subjected to 2D classification into 100 classes, of which seventeen with evident secondary structure and high signal-to-noise ratio were retained and exported to cryoSPARC v2.11 (Punjani et al., 2017); these seventeen 2D classes contained a total of 542948 particles. 3D heterogeneous refinement into seven classes then followed, these being seeded by low-pass filtered versions of 3D classes obtained from the in-house study of IGF-I-complexed IGF-1Rzip (data not shown). Of the seven 3D maps obtained, one displayed a structure similar to that reported for the IGF-I-bound holoreceptor (termed the "closed" conformation, from 108899 particles with a resolution of 6.78 Å), another displayed again a similar structure to that reported for the IGF-I-bound holoreceptor, but with the receptor legs significantly separated (termed herein the "open" conformation of the receptor, from 205471 particles and also with a resolution of 6.78 Å resolution), with the remaining five maps being judged as poor. 3D reconstruction then progressed by independent homogeneous refinement of the particles associated with the above open and closed conformation maps, yielding maps of resolution 4.10 Å and 4.61 Å, respectively. Independent local and non-uniform refinement of these sets then yielded maps of resolution 3.65 Å and 4.09 Å, respectively. At this stage it was clear that the domains of the IGF-1R and a single IGF-II could be docked into the maps in a fashion analogous to that within the insulin-complex IRΔβzip structure.

Such docking then enabled independent focused refinement of the "head" and "leg" volumes of both the open and closed conformations. The particles were then exported to RELION and subjected to CTF refinement and Bayesian "polishing" before a final refinement. The subtracted particles were refined to the final maps within cryoSPARC. The resultant resolution of the maps obtained were as follows: head volume of open conformation 3.21 Å, head volume of closed conformation 3.70 Å, legs volume of open conformation 4.26 Å and legs volume of closed conformation 4.21 Å. The above protocols and their output are summarized in Figures 2 and 3, with final statistical data provided in Tables 1 and S1. The two head volume maps appeared very similar; however, attempts to improve map resolution by combining the two underlying sets of particles did not succeed in producing a map of higher resolution that of Map^{HO}.

Model Building

Model building began by rigid-body docking (using UCSF Chimera (Pettersen et al., 2004)) of the individual receptor domains as extracted from PDB entry 5U8R (Xu et al., 2018) directly into a *B*-factor sharpened form of Map^{HO} (B_{sharp} = 47 Å^2, as implemented by cryoSPARC). IGF-II was modelled directly from IGF-I, based on the latter's structure as present in the co-crystal complex with the IGF-1R ectodomain (PDB entry 5U8Q) (Xu et al., 2018). Preliminary model improvement was then undertaken using COOT (version 0.8.9.1) (Emsley et al., 2010) and real-space-refinement within Phenix (version 1.16) (Afonine et al., 2018). This initial model was then used as targets for interactive flexible fitting using ISOLDE version 1.0b3 (Croll, 2018) starting from the biological apo-dimer derived from PDB entry 5U8R. Cα atoms from defined secondary structure components of the rigid-body docked individual domains were applied as weak positional restraints for their corresponding atoms in the starting model, while local geometry of each individual domain was maintained using a web of adaptive distance restraints. For each Cα, Cβ or Cγ atom (or their equivalent), the distance to each atom from the same group (excluding atoms from within the same residue) within an 8 Å sphere was restrained to its starting value using a potential function derived from a recent generalization of the Geman-McClure penalty scheme (Barron, 2019) to maintain local geometry while still allowing larger-scale deviations. Interactive tugging on selections of atoms was used where necessary to release the model from local minima. After this initial bulk fitting was accomplished, the Cα target restraints were released, as were the adaptive distance restraints on well-resolved regions. In domains where the resolution was insufficient to clearly resolve secondary structures, the adaptive distance restraints were maintained throughout the modelling process. All well-resolved regions were then interactively inspected and remodelled where necessary based on fit to the map and visual feedback from real-time Ramachandran and rotamer validation. These steps were then followed by further real-space refinement within Phenix iterated with manual model-building within COOT. During each real-space refinement run within Phenix, reference restraints to the input model (with strict rotamer matching) and secondary structure restraints were maintained throughout each cycle. Ramachandran and rotamer restraints were not imposed within Phenix, as the structure generated by ISOLDE was deemed sufficiently accurate with regard to both Ramachandran and rotamer statistics. Refinement was guided throughout by MolProbity statistics (Williams et al., 2018). The final model is termed here $Model^{HO}$.

Model building of the head domains corresponding to the closed-leg structure began by rigid-body placement (using Chimera) of $Model^{HO}$ directly into a *B*-factor sharpened form of Map^{HC} (B_{sharp} = 47 Å^2, as implemented by cryoSPARC). The model was then real-space refined within Phenix using (i) reference model restraints to $Model^{HO}$ (with strict rotamer matching) and (ii) secondary structure restraints, both in the absence of Ramachandran restraints or further rotamer restraints. The model generated by these steps is termed here $Model^{HC}$.

Model building of the leg domains of the open-leg structure began by manually-guided rigid-body docking (using Chimera) of two copies of domain FnIII-2 and a single copy of domain L1 and its associated αCT peptide directly into a *B*-factor sharpened form of Map^{LO} (B_{sharp} = 108 Å^2, as implemented by cryoSPARC). Density associated with the respective domains FnIII-3 and FnIII-3' was judged too poor to be modelled, even though the appropriately thresholded volumes associated with these domains appeared to have dimensions approximately corresponding to their crystallographic dimension. The model generated by these steps is termed here $Model^{LO}$.

Model building of the leg domains of the closed-leg structure began by manually-guided rigid-body docking (using Chimera) of two copies of domain FnIII-2 and of domain FnIII-3 and a single copy of domain L1 and its associated αCT peptide directly into a *B*-factor sharpened form of Map^{LC} (B_{sharp} = 66 Å^2, as implemented by CryoSPARC). The model generated by these steps is termed here $Model^{LC}$.

$Model^{HO}$, $Model^{LO}$, $Model^{HC}$ and $Model^{LC}$ were then re-inspected and further improved in ISOLDE, followed again by real-space refinement in Phenix subject to both input model and secondary structure restraints, again in the absence of Ramachandran or further rotamer restraints.

The final versions of $Model^{HO}$ and $Model^{LO}$ were then combined to form the "open-leg structure" of IGF-II bound to IGF-1Rzip and of $Model^{HC}$ and $Model^{LC}$ to form the "closed-leg structure" of IGF-II bound to IGF-1Rzip. Figures depicting these structures and their associated maps were generated using ChimeraX (Goddard et al., 2018).

Final statistics are presented in Tables 1 and S1.

Identification of Disulfide Links Using Mass Spectrometry (MS)

20 μg of the IGF-1R ectodomain construct IGF-1RΔβ (prepared as previously described (Whitten et al., 2009)) was resuspended in 6 M urea and 100 mM Tris-HCl pH 7.0 and subjected to protein digestion using a filter-aided sample preparation (FASP) column without cysteine disulfide-bond reduction and alkylation (Wisniewski et al., 2009). The peptide solution was acidified (0.1% formic

acid) and lyophilized using a SpeedVac AES 1010 (ThermoFisher Scientific). Peptides were injected and separated by reversed-phase liquid chromatography on a M-class UPLC system (Waters; Milford, Massachusetts) using a 250 mm × 75 μm column (1.6 μm C18, packed emitter tip; Ion Opticks, Parkville, Australia) with a linear 90-min gradient at a flow rate of 400 nL.min^{-1} from 98% solvent A (0.1% formic acid in Milli-Q water) to 35% solvent B (0.1% formic acid, 99.9% acetonitrile). The nano-UPLC was coupled on-line to a Q-Exactive Orbitrap mass spectrometer equipped with a nano-electron spray ionization source (ThermoFisher Scientific). The Q-Exactive was operated in a data-dependent mode, switching automatically between one full-scan and subsequent MS/MS scans of the ten most abundant peaks. The instrument was controlled using Exactive series version 2.1 build 1502 (ThermoFisher Scientific) and Xcalibur 3.0 (ThermoFisher Scientific). Full-scans (m/z 350–1,850) were acquired with a resolution of 70,000 at 200 m/z. The ten most intense ions were sequentially isolated with a target value of 10000 ions and an isolation width of 3 m/z and fragmented using HCD with NCE of 27. Maximum ion accumulation times were set to 50 ms for full MS scan and 200 ms for MS/MS.

Raw files were analysed using MaxQuant version 1.6.7.0 (Cox and Mann, 2008). The database search was performed using the UNIPROT *Homo sapiens* database plus common contaminants with strict trypsin specificity allowing up to 2 missed cleavages. MaxQuant APL files were converted to MGF files using the APL to MGF converter software (https://www.wehi.edu.au/people/andrew-webb/1298/apl-mgf-converter). Cysteine crosslinked peptides were identified from the MGF files using StavroX software version 3.6.6.5 (Götze et al., 2012). Trypsin was set as the enzyme allowing for three missed cleavages at lysines and arginines. Precursor precision was set at 10 ppm with fragment ion precision set at 20 ppm. Spectra associated with the Cys662-Cys662’ and Cys633-Cys849 disulfide bonds are presented in Figure S6.

QUANTITATION AND STATISTICAL ANALYSIS

Ligand affinity data were analysed as described in the “Receptor competition binding assay” section of the Methods Details using the software package GraphPad detailed in the Key Resources Table.

CryoEM single-particle reconstructions and associated model building were performed as described in the “Three-dimensional single-particle reconstruction” and “Model building” sections of the Methods Details using the software packages RELION, CryoSPARC, ISOLDE, Phenix, Coot, Chimera and ChimeraX detailed in the Key Resources Table.

Mass spectroscopy data were analysed as described in the “Identification of disulfide links using mass spectrometry (MS)” section of the Methods Details using the software packages MaxQuant, StavroX, Exactive and Xcalibur detailed in the Key Resources Table, with further detail provided in Figure S6.

Structure, Volume *28*

Supplemental Information

How IGF-II Binds to the Human Type 1

Insulin-like Growth Factor Receptor

Yibin Xu, Nicholas S. Kirk, Hariprasad Venugopal, Mai B. Margetts, Tristan I. Croll, Jarrod J. Sandow, Andrew I. Webb, Carlie A. Delaine, Briony E. Forbes, and Michael C. Lawrence

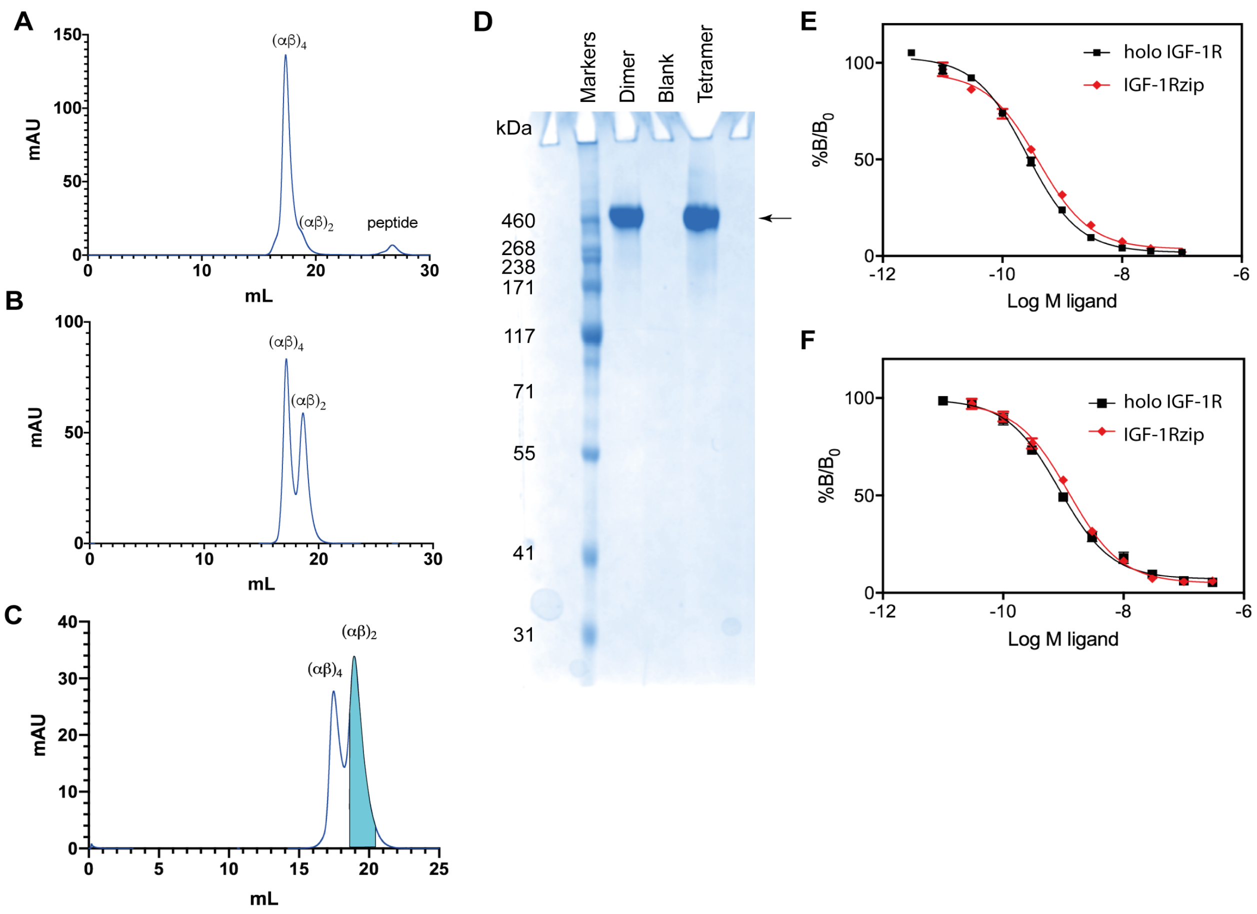

FIGURE S1. Purification and characterization of IGF-1Rzip. Related to STAR methods sections 'Purification of IGF-1Rzip' and 'Receptor competition binding assays'. (**A**) First size-exclusion chromatograph showing a major peak corresponding to the tetrameric form of IGF-1Rzip with a peak shoulder representing the dimeric form of IGF-1Rzip. (**B**) Second size-exclusion chromatograph obtained from the $(\alpha\beta)_2$ shoulder fractions of (A) showing enhanced separation of the dimeric and tetrameric form of IGF-1Rzip. (**C**) Final size-exclusion chromatograph obtained from the $(\alpha\beta)_2$ shoulder fractions of (B) showing further separation of the dimeric and tetrameric form of IGF-1Rzip; fractions highlighted in cyan indicate final pooled fractions of predominantly dimeric protein used for cryoEM analysis in complex with IGF-II. (**D**) Coomassie-stained non-reducing SDS-PAGE gel of the pooled dimeric fractions and pooled tetrameric fractions of IGF-1Rzip from (B) obtained under non-reducing conditions, showing a high level of product purity and indicating that the tetrameric form of the protein product is a non-disulfide-linked dimer of disulfide-linked monomers. The lanes to the left of the "Markers" and right of the "Tetramer" lanes are blank; further lanes cropped beyond these are unrelated to this study. (**E**) Labelled-IGF-I displacement assay comparing IGF-I affinity for IGF-1Rzip (n=9) and for holo IGF-1R (n=9; single individual measurement omitted as aberrant). (**F**) Labelled-IGF-II displacement assay comparing IGF-II affinity for IGF-1Rzip (n=9; four individual measurements omitted as aberrant) and for holo IGF-1R (n=6). For (E) and (F): data are represented as mean ± SEM and, where not visible, error bars are smaller than the marker size.

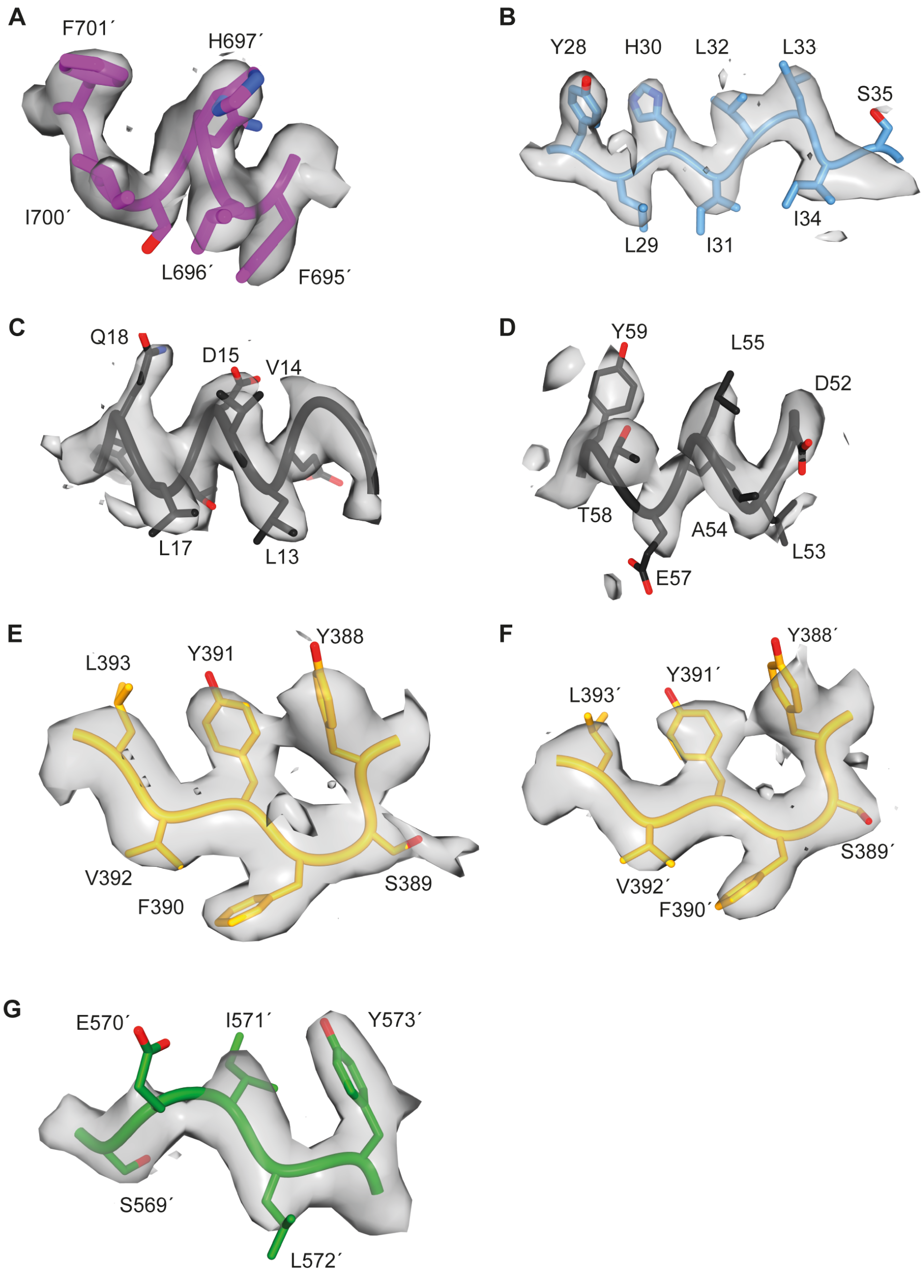

FIGURE S2. Map^{HO} (B_{sharp}= 47.1 Å^2) **and associated atomic model. Related to Figure 4.** (**A**) αCT´ residues Tyr695´ to Val701´. (**B**) Domain L1 residues Tyr28 to Ser35. (**C**) IGF-II B-domain helix residues Val10 to Gly19. (**D**) IGF-I A-domain residues Asp52 to Tyr59. (**E**) Domain L2 residues Ser389 to Leu393. (**F**) Domain L2´ residues Ser389´ to Leu393´. (**G**) Domain F1´ residues Ser569´ to Tyr573´. Surface contours are drawn at a common level of 0.7 units and restricted to lie with 2.5 Å of the associated model segment.´

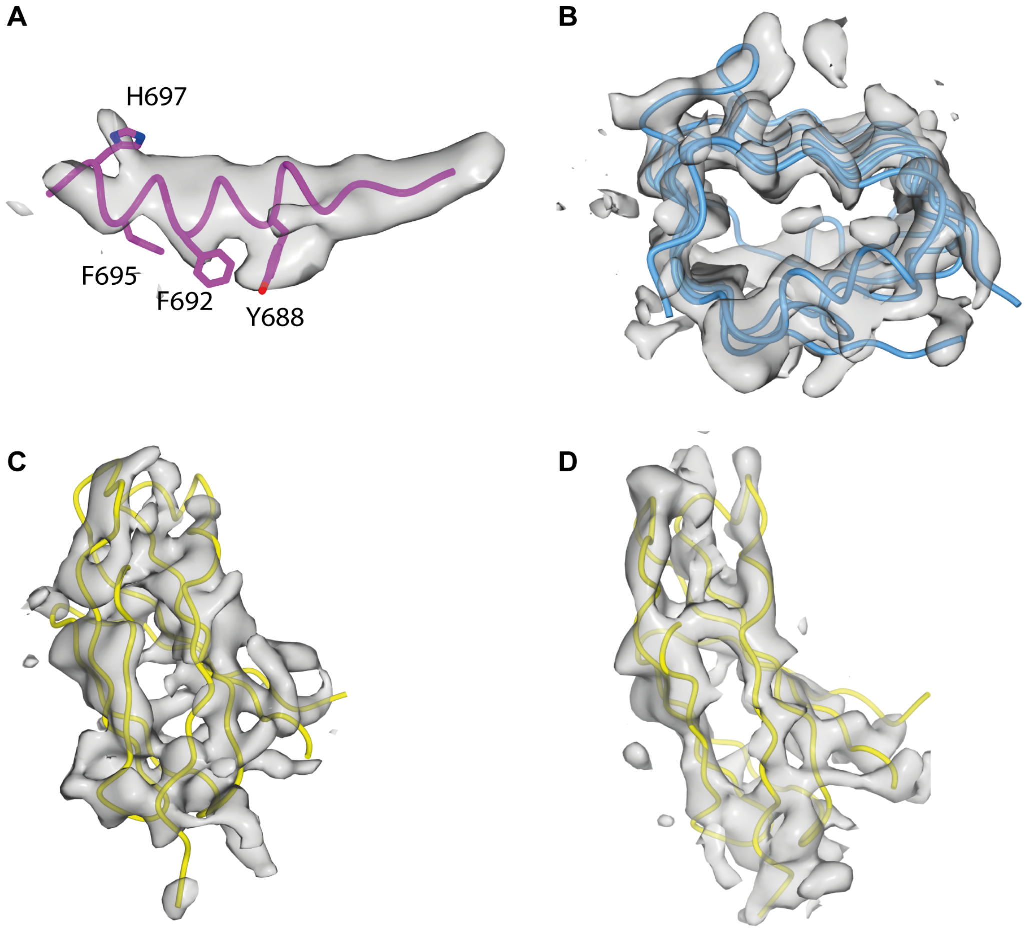

FIGURE S3. Map^{LO} (B_{sharp}= 104.7 Å^2) **and associated atomic model. Related to Figure 4.** (**A**) αCT residues 680-698. (**B**) Domain L1´, residues 1´-150´. (**C**) Domain FnIII-2´, residues 585´-622´ + 720´-800´. (**D**) Domain FnIII-2, residues 585-622 + 720-800. Surface contours are drawn at a common level of 0.5 units and restricted to lie with 3.0 Å of the associated model segment.

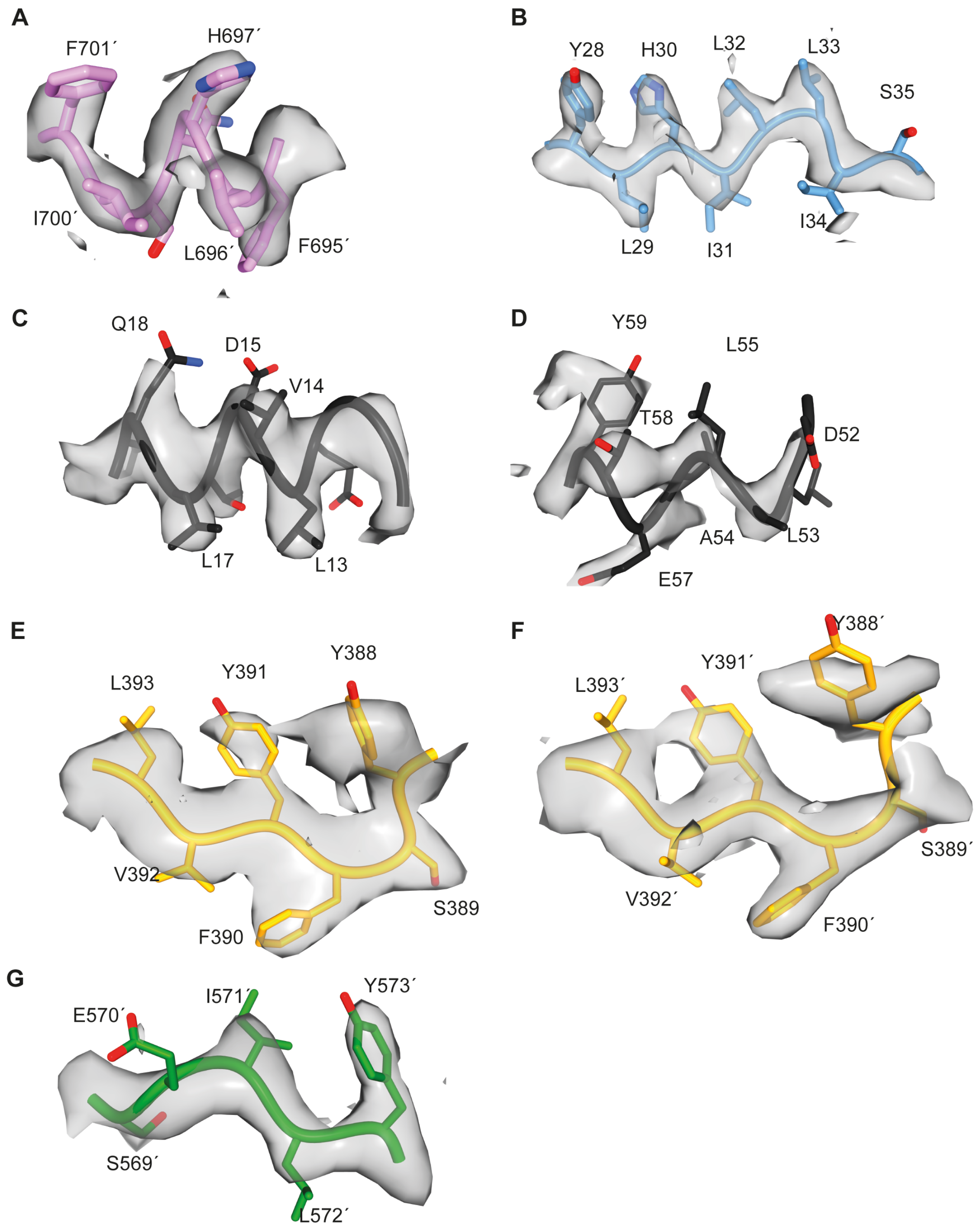

FIGURE S4. Map^HC (B_{sharp}= 47.7 Å^2) and associated atomic model. Related to Figure 4. (**A**) αCT´ residues Tyr695´ to Val701´. (**B**) Domain L1 residues Tyr28 to Ser35. (**C**) IGF-II B-domain helix residues Val10 to Gly19. (**D**) IGF-I A-domain residues Asp52 to Tyr59. (**E**) Domain L2 residues Ser389 to Leu393. (**F**) Domain L2´ residues Ser389´ to Leu393´. (**G**) Domain F1´ residues Ser569´ to Tyr573´. Surface contours are drawn at a common level of 0.7 units and restricted to lie with 2.5 Å of the associated model segment.

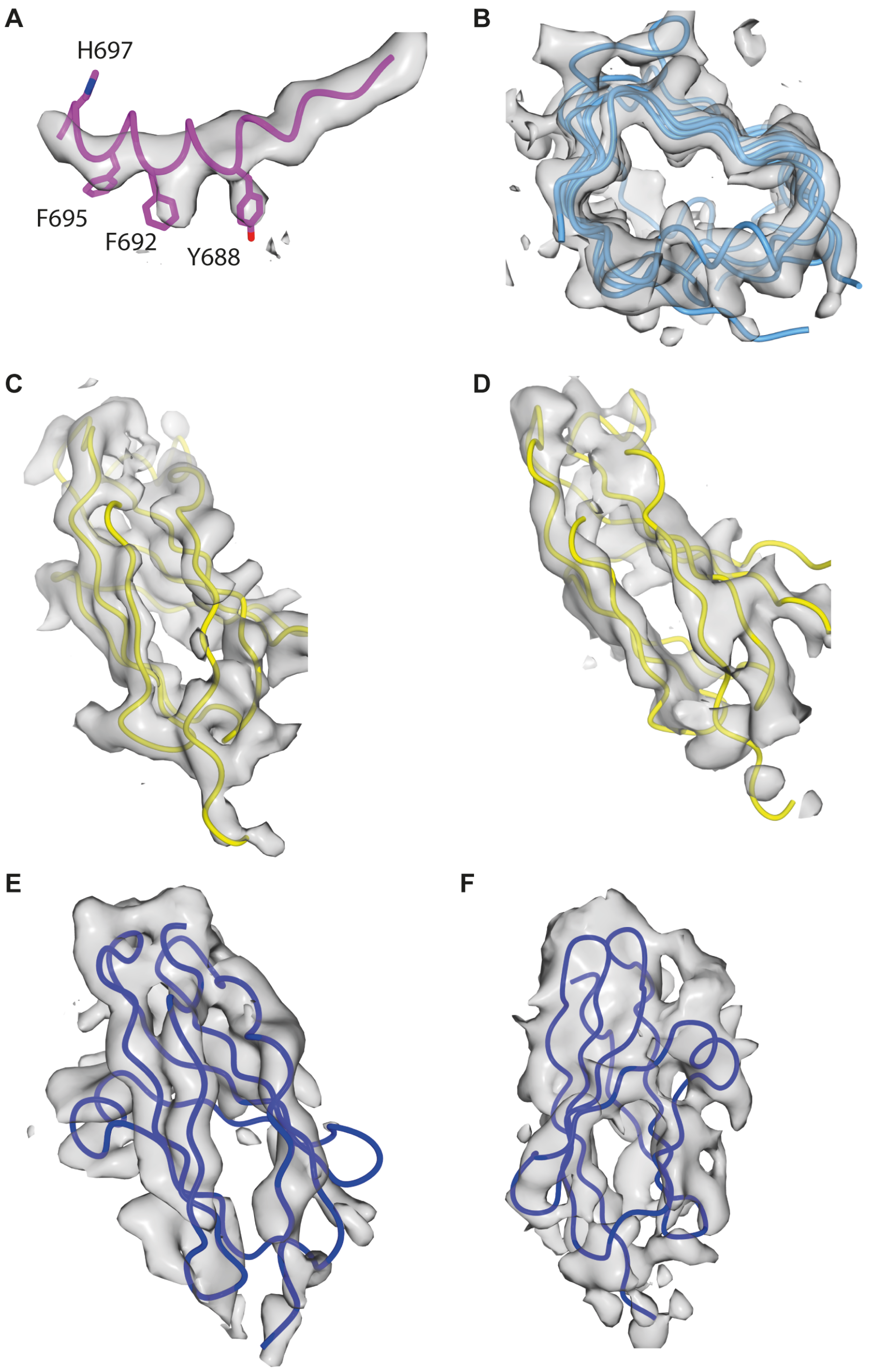

FIGURE S5. Map^{LC} (B_{sharp}= 66.4 Å^2) **and associated atomic model. Related to Figure 4.** (**A**) αCT residues 680-698. (**B**) Domain L1´, residues 1´-150´. (**C**) Domain FnIII-2´, residues 585´-622´ + 720´-800´. (**D**) Domain FnIII-2, residues 585-622 + 720-800. (**E**) Domain FnIII-3´, residues 585´-622´ + 720´-800´. (**F**) Domain FnIII-3, residues 585-622 + 720-800. Surface contours in panels (A-D) are drawn at a common level of 0.5 units and in panels (E,F) at a common level of 0.33 units. All contours restricted to lie with 3.0 Å of the associated model segment.

A

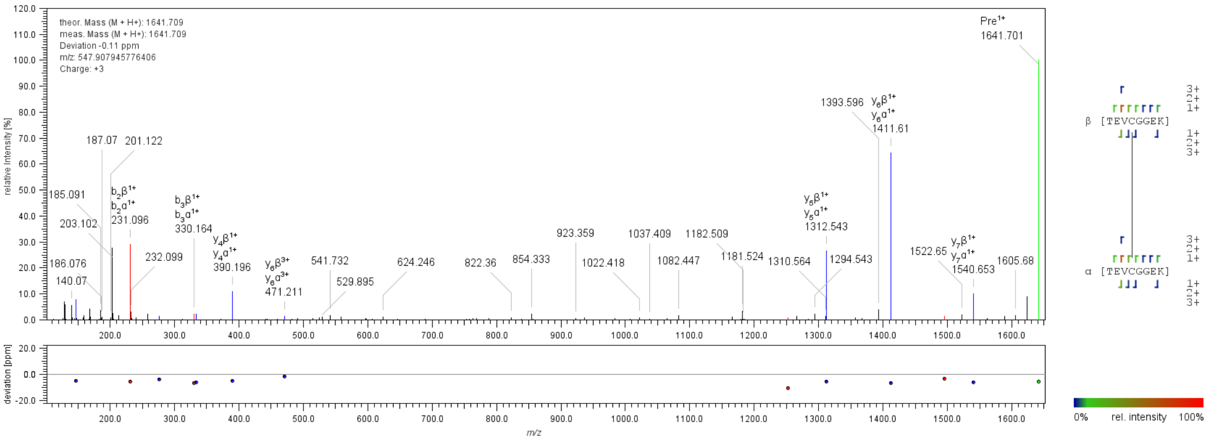

B

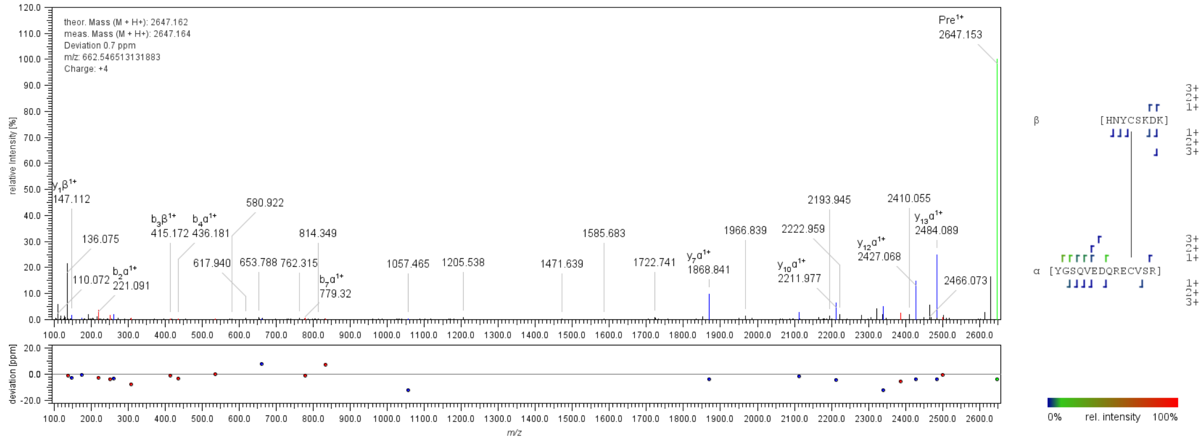

FIGURE S6. Mass spectroscopic identification of peptidic fragments of the IGF-1R ectodomain construct IGF-1RΔβ. Related to STAR Methods section 'Identification of disulfide links using mass spectrometry (MS)'. (**A**) Cys662-Cys662´. (**B**) Cys633-Cys849 (equivalently, Cys633´-Cys849´).

TABLE S1. Residues included in the open- and closed-leg structures of IGF-1Rzip.IGF-II. Related to Figure 4.

PDB	Conformation	Region	Map	Receptor*		IGF-II
				αβ	(αβ)´	
6VWG	Open-leg	Head	Map^{HO}	1-155<br>161-257<br>266-293<br>299-457	299´-511´<br>518´-578´<br>673´-705´	5-32<br>37-62
6VWH	Open-leg	Legs	Map^{LO}	577-633<br>682-704<br>744-799	1´-37´<br>41´-154´<br>162´-300´<br>577´-633´<br>744´-799´	-
6VWI	Closed-leg	Head	Map^{HC}	1-155<br>161-257<br>266-293<br>299-457	299´-511´<br>518´-578´<br>673´-705´	5-32<br>37-62
6VWJ	Closed-leg	Legs	Map^{LC}	580-642<br>682-704<br>744-897	1´-37´<br>41´-154´<br>162´-300´<br>580´-642´<br>744´-897´	-

* The αβ monomer of IGF-1Rzip is defined as that which contributes domain L1 to the IGF-II binding site; the (αβ)´ monomer is that whose domain L1 is ligand-free within this structure.
